# Identification of a novel ferroptosis-related gene signature associated with prognosis, the immune landscape, and biomarkers for immunotherapy in ovarian cancer

**DOI:** 10.3389/fphar.2022.949126

**Published:** 2022-10-25

**Authors:** Yilong Liu, Suya Du, Mengying Yuan, Xia He, Changyu Zhu, Ke Han, Yuyan Zhu, Qianwen Yang, Rongsheng Tong

**Affiliations:** ^1^ Department of Pharmacy, Sichuan Academy of Medical Science and Sichuan Provincial People’s Hospital, School of Medicine, University of Electronic Science and Technology of China, Chengdu, China; ^2^ Department of Clinical Pharmacy, Sichuan Cancer Hospital and Institute, Sichuan Cancer Center, School of Medicine, University of Electronic Science and Technology of China, Chengdu, China; ^3^ Personalized Drug Therapy Key Laboratory of Sichuan Province, School of Medicine, University of Electronic Science and Technology of China, Chengdu, China

**Keywords:** ovarian cancer, ferroptosis, biomarker, prognosis, immunotherapy

## Abstract

Ferroptosis has been implicated in tumor progression and immunoregulation. Identification of ferroptosis-related prognostic gene is important for immunotherapy and prognosis in ovarian cancer (OV). We assessed the potential predictive power of a novel ferroptosis-related gene (FRG) signature for prognosis and immunotherapy in Asian and Caucasian OV populations. We collected gene expression profiles and clinicopathological data from public databases. The least absolute shrinkage and selection operator Cox regression algorithm was used to construct the FRG signature. Receiver operating characteristic (ROC) curve, Kaplan-Meier method, Cox regression model were used to evaluate the clinical benefits of FRG signature. Gene functional and gene set enrichment analyses were used for functional annotation and immune landscape analysis. A 15-FRG signature was constructed and used to stratify patients into two risk groups. Patients in the high-risk group had significantly worse survival. The risk score was a significant independent risk factor for OS. The area under the ROC curve indicated the good prediction performance of the FRG signature. Notably, the low-risk group showed a significant enrichment in immune-related pathways and a “hot” immune status. The risk score was found to be an efficient and robust predictor of response to immunotherapy. In conclusion, our study identified a novel 15-FRG prognostic signature that can be used for prognostic prediction and precision immunotherapy in Asian and Caucasian OV populations.

## Introduction

Ovarian cancer (OV), the third most common gynecologic malignancy and the second leading cause of cancer-related deaths (Bray et al., 2018), is frequently diagnosed late due to hidden and nonspecific symptoms in the early stage, resulting in a 5-year survival rate of only 47% after diagnosis, which is low in comparison with the 85% survival rate of breast cancer (Lheureux et al., 2019). The standard therapy for OV relies heavily on upfront surgical debulking followed by platinum-based chemotherapy (Lheureux et al., 2019), with a favorable early response observed in approximately 80% of patients. However, unfortunately, the disease soon recurs in most of these patients. Meanwhile, OV is a highly heterogeneous disease that comprises multiple histological subtypes and different microenvironmental features (Chen et al., 2018; Geistlinger et al., 2020). Studies have shown that there are huge differences in the treatment effect and prognosis of individuals with OV (Morand et al., 2021), which make the prediction of tumor treatment response and prognosis challenging. Therefore, considering the high recurrence rate and cellular heterogeneity of OV, the development of innovative treatments and refinement of prognostic prediction are urgently needed.

Ferroptosis is a novel iron-dependent form of non-apoptotic regulated cell death, with distinct features of overaccumulation of reactive oxygen species (ROS) and lipid peroxidation (Yu et al., 2017; Tang et al., 2019). Recently, ferroptosis induction has been demonstrated as a potential prevention or therapeutic modality in various diseases (Tang et al., 2018; Qiu et al., 2020), especially for anticancer treatments (Xu et al., 2019; Wang et al., 2020). In addition to small-molecule inhibitors and agonists, various ferroptosis-related genes (FRGs) have been identified as drivers, suppressors, and markers in OV, including *CYBB* and *TAZ* (Yang W. et al., 2020), which have been confirmed as ferroptosis-driving factors that contribute to the sensitization of OV cells to ferroptosis. In contrast, *GPX4*, *GCH1*, and *FSP1* (Li et al., 2021) mediate three distinct mechanisms of ferroptosis protection to ensure tumor cellular homeostasis. And, a previous study has indicated that inducing ferroptosis is correlated with prolonged progression free survival in patients with platinum-resistant ovarian cancer (Chekerov et al., 2018). Taken together, these compelling findings demonstrate that OV may be highly sensitive to ferroptosis, and targeting ferroptosis may be helpful to improve the prognosis of OV. However, the underlying ferroptosis-related prognostic biomarkers in OV remain largely unknown. Hence, it is important to excavate more ferroptosis-related biomarkers for treatment and prognosis in OV. Up to now, several studies have extensively explored the relationship between FRGs and tumor prognosis, and constructed a prognostic signature for a variety of tumor types, such as a novel 10-FRG prognostic signature in liver cancer, a novel 9-FRG prognostic signature in breast cancer (Liang et al., 2020; Wang D. et al., 2021). Regretfully, most previous studies have failed to verify these relationships across multiple regions and races, which may lead to weak generalization ability of research results among different races.

Thus, in the present study, the prognostic capacity of FRGs in patients with OV was comprehensively analyzed using publicly available gene expression profiles obtained from the Asian and Caucasian populations. Besides, a prognostic signature was constructed and its potential associations with the immune landscape and immunotherapy were explored. Notably, our results revealed the prognostic value of a novel 15-FRG signature and provided a promising predictor of response to immunotherapy and chemotherapy.

## Materials and methods

### Public data collection

The GSE32062 microarray dataset, comprising the data and clinical information of 260 OV patients, was obtained from the Gene Expression Omnibus (GEO) website (http://www.ncbi.nlm.nih.gov/geo) for use as a training cohort. The raw expression data were background-corrected by applying the “normexp” method (with an offset of 1) and were subjected to quantile normalization using the “limma” R package. When genes were mapped to more than one probe, the gene with the mean fold-change value was selected.

International Cancer Genome Consortium (ICGC) (OV-AU) RNA-sequencing (RNA-seq) data of 93 OV patients and their clinical information were obtained from the ICGC data portal (https://dcc.icgc.org/projects/OV-AU) for use as an external validation cohort. RNA-seq datasets of 41 patients treated with anti-PD-1 monotherapy (Gide et al., 2019) and 42 patients treated with anti-CTLA4 monotherapy (Van Allen et al., 2015) were obtained from the Tumor Immune Dysfunction and Exclusion (TIDE) website (http://tide.dfci.harvard.edu/download/) for use as an external validation cohort. All the datasets used for analysis are freely and publicly available, hence local ethical approval was exempted.

FRGs were retrieved from a public ferroptosis database (FerrDb; http://www.zhounan.org/ferrdb/) (Zhou and Bao, 2020) and were limited to published human studies. Immune-related genes (IRGs) were retrieved from a public immunology database (ImmPort; https://www.immport.org/shared/genelists) (Bhattacharya et al., 2018). A comprehensive gene list is provided in Supplementary Tables S1A, S1B.

### Ferroptosis-related gene signature construction and validation

Univariate analyses for identifying overall survival (OS)-related FRGs were performed using Cox regression analysis, with adjusted (adj.) *p* < 0.05 considered significant. The STRING v11.0 database (Szklarczyk et al., 2010) (https://string-db.org/) was used for protein-protein interaction (PPI) analysis. The least absolute shrinkage and selection operator (LASSO) algorithm was used to penalize the risk of overfitting and to construct a Cox regression model with an optimal penalty parameter λ selected based on a 10-fold cross validation (Simon et al., 2011). The FRG signature was constructed as follows: the risk score = ∑ (β × m), where β is the Cox coefficient and m is the z-score standardized expression value of the corresponding gene. According to the median risk score value, patients were categorized into high- and low-risk groups. Kaplan–Meier (KM) survival curve analysis was conducted in combination with a log-rank test, using the “survival” and “survminer” packages. The predictive performance of the risk score was determined with a time-dependent receiver operating characteristic (ROC) curve, using the R package “timeROC.” The prognostic value was validated in the external Caucasian validation cohort.

### Validation of independent prognostic role

The risk score and other available clinical variables were included for univariate analyses using the Cox proportional hazard model. Variables that were significant (*p* < 0.05) in univariate analyses were considered for multivariate analyses.

### Construction and validation of a predictive nomogram

Nomograms are widely applied to simplify statistical prediction models into an objective and operational graphical tool (Iasonos et al., 2008). All available prognostic factors were selected to build a nomogram to investigate the probability of 2-, 4-, and 6-year OS of patients with OV. Subsequently, the nomogram was validated by discrimination and calibration. Discrimination was measured with the concordance index (C-index), using the R package “survcomp.” Calibration was evaluated graphically by plotting the nomogram prediction probabilities against the actual proportion. Overlap with the slash diagonal indicates that the model is completely consistent.

### Functional enrichment analysis

To investigate the underlying biological functions of the FRG signature, 15 FRGs and differentially expressed genes (DEGs) with adj. *p* < 0.05 and |log2FC| > 0.5 between the high- and low-risk samples were selected separately for Gene Ontology (GO) classification and Kyoto Encyclopedia of Genes and Genomes (KEGG) pathway analyses, using the R packages “limma” and “clusterProfiler.” Gene set enrichment analysis (GSEA) of the high- and low-risk samples was conducted using the GSEA software (Subramanian et al., 2005) (http://www.broadinstitute.org/gsea).

### Immune infiltration landscape analysis

The enrichment scores of various immune cells represented by 16 gene sets and immune-related functions represented by 13 gene sets were quantified using single-sample gene (ss)GSEA in the Bioconductor package “GSVA” (Barbie et al., 2009; Rooney et al., 2015). The annotated immune-related gene sets are provided in Supplementary Table S1C. Enrichment scores for seven steps of the cancer-immunity cycle were calculated with the Tracking Tumor Immunophenotype (TIP) meta-server tool (http://biocc.hrbmu.edu.cn/TIP/) (Xu et al., 2018). The Estimation of STromal and Immune cells in MAlignant Tumor tissues using Expression data (ESTIMATE) immune score was computed to analyze the infiltration levels of immune cells using the “ESTIMATE” R package. The expression correlation between the key immune checkpoint molecules and risk score were further analyzed.

### Validation of the predictive power of the risk score in immunotherapy and chemotherapy

SubMap method from GeneParttern was applied to predict the response to immunotherapy in OV patients with high- and low-risk groups (Hoshida et al., 2007). Besides, the anti-PD-1 and anti-CTLA4 cohorts were used as the independent external validation cohorts, which were also used on other predictors to compare with our signature in terms of performance. Fourteen published predictors of clinical response to immune checkpoint blockade (ICB), such as the TIDE score, microsatellite instability score (MSI), and T-cell exclusion score, were integrated into TIDE computational framework (Fu et al., 2020) (http://tide.dfci.harvard.edu). Prediction scores were calculated and downloaded for each patient in the anti-PD-1 and anti-CTLA4 cohorts after uploading the normalized gene expression profiles to TIDE. Furthermore, the pRRophetic algorithm was utilized to calculate the half-maximal inhibitory concentration (IC50) values for monitoring the response of chemotherapy to the ovarian cancer patients (Geeleher et al., 2014).

### Statistical analysis

All statistical analyses were carried out using the R programming software (version 3.6.3). Two-sided Pearson’s chi-squared test or Fisher’s exact test was used for categorical data, and the non-parametric Wilcoxon test was applied for quantitative data. KM analysis with the log-rank test for OS was conducted using the auto-select best threshold or the median value. Correlations between variables were determined using Spearman or Pearson correlation. Significant differences between two correlated ROC curves were analyzed using DeLong’s test. Unless noted otherwise, *p* < 0.05 was considered statistically significant.

## Results

**FIGURE 1:**
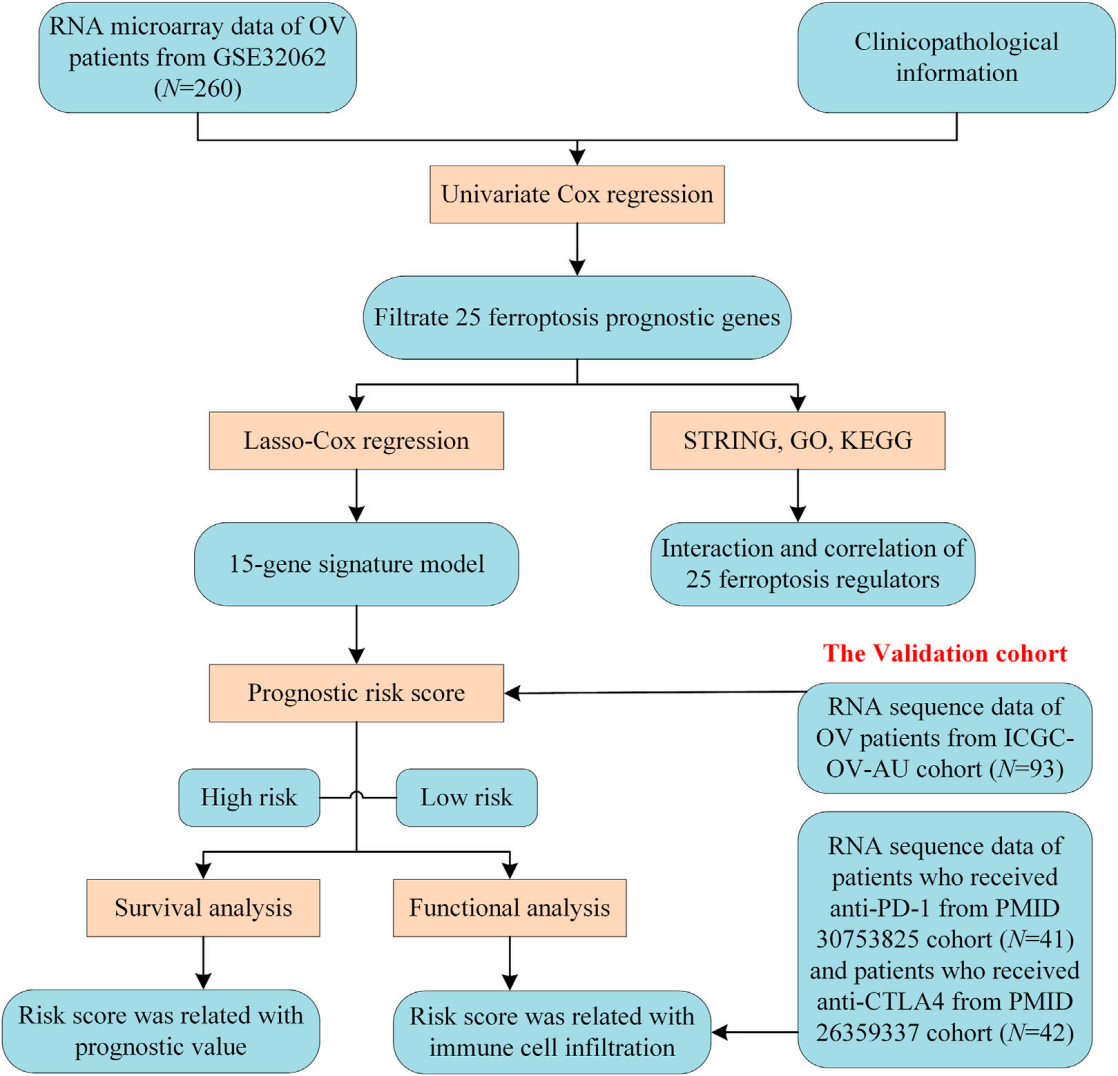
Flow chart for cohort collection and bioinformatics analysis.

A methodology flow chart for the present study is shown in Figure 1. In total, 260 Asian OV samples from the GSE32062 cohort and 93 Caucasian OV samples from the ICGC (OV-AU) cohort with complete clinical information were included. Detailed clinical information for these samples is provided in Supplementary Table S2A.

### Identification of prognostic ferroptosis-related genes in the GSE32062 cohort

**FIGURE 2:**
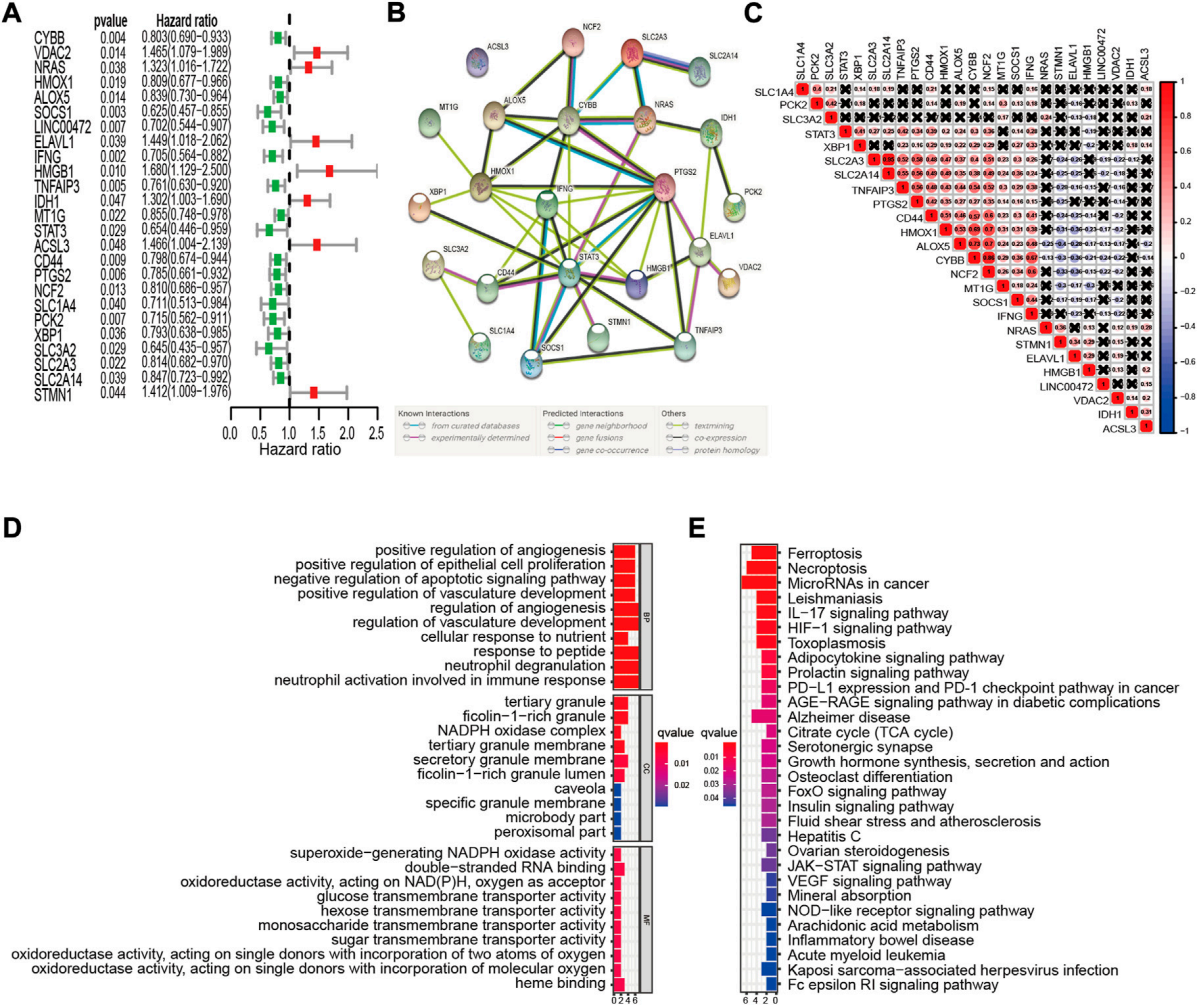
Identification of prognostic FRGs in the GSE32062 cohort. **(A)** Forest plots of HRs and 95% CIs of the association between FRGs expression and OS. **(B)** A PPI network showing the interactions among the prognostic FRGs. **(C)** FRGs correlation network. **(D,E)** The most significantly enriched GO terms **(D)** and KEGG pathways **(E)** are displayed. FRG, ferroptosis-related gene; HR, hazard ratio; CI, confidence interval; OS, overall survival; PPI, protein-protein interaction; GO, Gene Ontology; KEGG, Kyoto Encyclopedia of Genes and Genomes.

We analyzed 213 well-defined FRGs in this study, namely 75 ferroptosis driver genes, 58 ferroptosis suppressor genes, and 80 ferroptosis marker genes. Detailed information on these FRGs is provided in Supplementary Table S1A. The relationship between FRG expression levels and the OS of patients in the GSE32062 cohort was evaluated using univariate Cox proportional hazards regression analysis. Twenty-five FRGs were significantly correlated with OS (*p* < 0.05, Figure 2A). Among the 25 prognostic FRGs, seven genes (*IDH1*, *NRAS*, *STMN1*, *ELAVL1*, *VDAC2*, *ACSL3*, and *HMGB1*) were identified as risk factors, with hazard ratios (HRs) > 1. The remaining 18 genes (*SOCS1*, *SLC3A2*, *STAT3*, *LINC00472*, *IFNG*, *SLC1A4*, *PCK2*, *TNFAIP3*, *PTGS2*, *XBP1*, *CD44*, *CYBB*, *HMOX1*, *NCF2*, *SLC2A3*, *ALOX5*, *SLC2A14*, and *MT1G*) were identified as protective factors, with HRs < 1. In a PPI network of the prognostic FRGs, *PTGS2*, *STAT3*, *HMOX1*, *IFNG*, and *CYBB* were hub genes (Figure 2B). Furthermore, most of the protective factors were strongly positively correlated with each other (*p* < 0.05, Figure 2C). GO enrichment analysis revealed that the 25 FRGs were primarily involved in the biological process of tumor progression, including “positive regulation of angiogenesis,” “epithelial cell proliferation,” “negative regulation of apoptotic signaling pathway,” and other immune-specific processes, including “neutrophil degranulation” and “neutrophil activation involved in immune response” (adj. *p* < 0.05, Figure 2D). KEGG analysis results suggested that these genes were correlated with ferroptosis and necroptosis (adj. *p* < 0.05, Figure 2E).

### Construction of a novel 15-ferroptosis-related gene prognostic signature in the GSE32062 cohort

**FIGURE 3:**
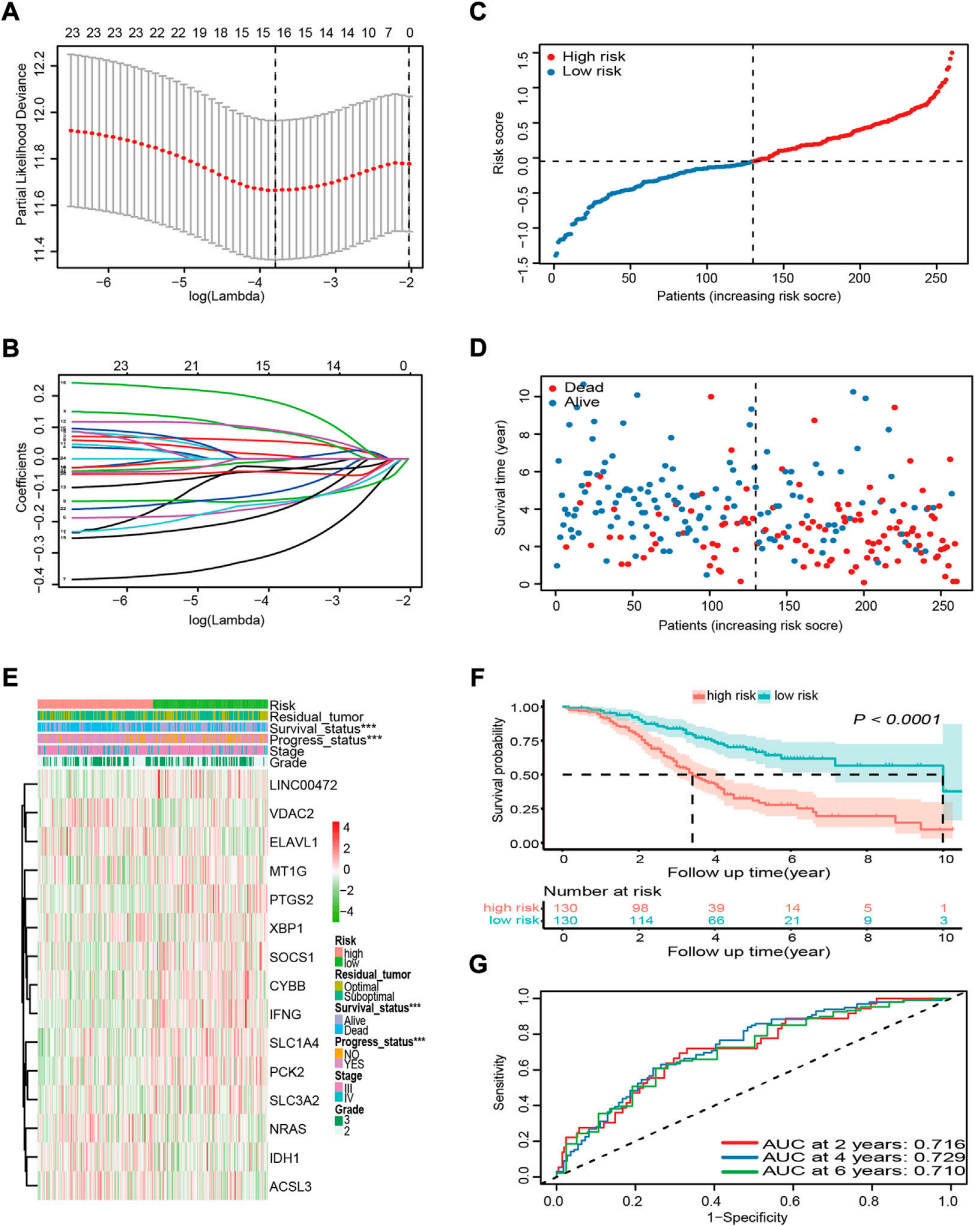
Construction and prognostic analysis of the 15-FRG prognostic signature based on the GSE32062 cohort data. **(A)** Penalty parameter λ optimization using the LASSO algorithm, with 10-fold cross validation. The optimal values using minimum criteria (left) and the one standard error of the minimum criteria (right) are indicated by dotted vertical lines. **(B)** LASSO coefficient profiles of the 25 candidate genes. **(C)** Distribution and median values of the risk scores. **(D)** Distributions of survival time, survival status, and risk score. **(E)** Distributions of risk score, clinical characteristics, and gene expression panels. *p < 0.05; **p < 0.01; ***p < 0.001. **(F)** Kaplan–Meier curves for OS in the different groups. **(G)** Time-dependent ROC curves and their AUCs verifying the prediction efficacy of the risk score. FRG, ferroptosis-related gene; LASSO, least absolute shrinkage and selection operator; OS, overall survival; ROC, receiver operating characteristic; AUC, area under the curve.

Genes without prognostic significance were filtered out, leaving 25 genes for further analysis. It is well known that the more genes the signature included, the more complex it became. Hence, the LASSO algorithm was employed to shrink the variables and to optimize the signature. A 15-FRG signature was constructed based on the optimal λ value (Figures 3A,B). Based on the expression levels of the 15 FRGs and the corresponding regression coefficients, the following signature formula was built: risk score = −0.0291 × *CYBB* + 0.0548 × *VDAC2* + 0.0836 × *NRAS* – 0.1457 × *SOCS1* – 0.2943 × *LINC00472* + 0.0101 × *ELAVL1* – 0.1289 × *IFNG* + 0.0873 × *IDH1* – 0.0351 × *MT1G* + 0.1742 × *ACSL3* – 0.1379 × *PTGS2* – 0.1599 × *SLC1A4* – 0.0432 × *PCK2* – 0.0044 × *XBP1* − 0.1017 × *SLC3A2*. To evaluate the prognostic prediction performance of the 15-FRG signature, the risk score of each sample was calculated according to the signature formula above. The OV patients were classified into a high-risk group (*n* = 130) and a low-risk group (*n* = 130) based on median risk score (Figure 3C). Patients with a high risk of the disease tended to suffer from earlier disease progression and worse survival status than their low-risk counterparts (*p* < 0.001, Figures 3D,E). Meanwhile, KM survival analysis revealed that the OS rate was significantly worse in the high-risk group compared with the low-risk group (*p* < 0.0001, Figure 3F). To exclude the influence of confounding clinical characteristics on the risk score, we further stratified patients by clinical variables to evaluate the prognostic prediction performance of the risk score on OS. The results reconfirmed that the risk score could classify the patients into high- and low-risk groups with significantly different OS rate (Supplementary Figures S1A–C). The prognostic performances of the 15 ferroptosis-related genes were also further confirmed by using Kaplan Meier plotter online tool. The results revealed that, apart from ELAVL1 and LINC00472, the other 13 FRGs were also significantly associated with patient OS (Supplementary Figure S2). Based on our findings above, time-dependent ROC curves were constructed to further evaluate the accuracy of the risk score for predicting prognosis. The area under the ROC curve (AUC) of the risk score for OS reached 0.716, 0.729, 0.710 at 2, 4, 6 years respectively, which suggested that the risk score had adequate prediction efficiency (Figure 3G).

### Validation of the 15-ferroptosis-related gene prognostic signature in the international cancer genome consortium cohort

**FIGURE 4:**
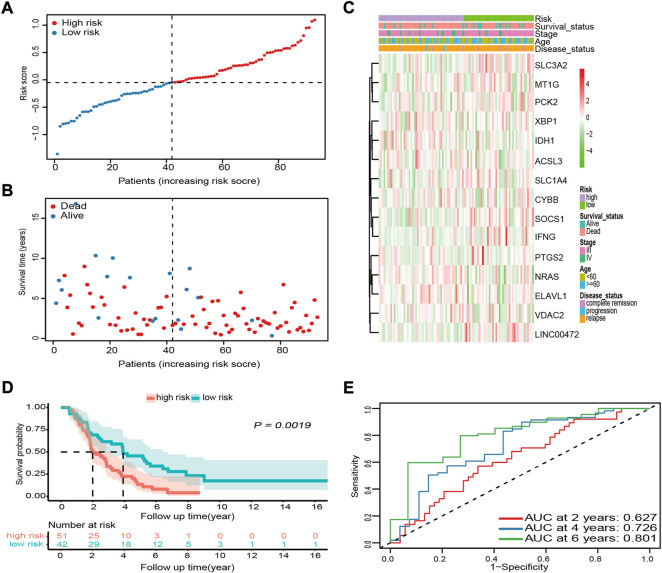
External validation of the prognostic performance of the 15-FRG signature in the ICGC cohort. **(A)** Distribution and median values of the risk scores. **(B)** Distributions of survival status, survival time, and risk score. **(C)** Distributions of risk score, clinical characteristics, and gene expression panels. **(D)** Kaplan–Meier curves for OS in the different groups based on the cut-off point determined for the GSE32062 cohort. **(E)** Time-dependent ROC curves and their AUCs. FRG, ferroptosis-related gene; ICGC, International Cancer Genome Consortium; OS, overall survival; ROC, receiver operating characteristic; AUC, area under the curve.

To validate the robustness and reproducibility of the signature constructed using Asian cohort data for predicting OS in Caucasian cohort data, we first calculated the risk score for each Caucasian OV sample in the ICGC cohort with the formula used in the GSE32062 cohort. Based on the median risk score of the GSE32062 cohort, patients from the ICGC cohort were divided into high- and low-risk groups (Figure 4A). As expected, similar results were obtained in Caucasian OV patients. In the high-risk group, patients were more likely to die earlier (Figure 4B) and the risk of mortality was higher (*p* = 0.0019, Figures 4C,D). Comparison of OS stratified by age, tumor stage, and disease status also further confirmed these results (Supplementary Figures S1D–F). Further, the AUC of the risk score for OS was 0.627 at 2 years, 0.726 at 4 years, and 0.801 at 6 years in the Caucasian cohort, suggesting that the risk score had adequate generalization performance between different races (Figure 4E).

**FIGURE 5:**
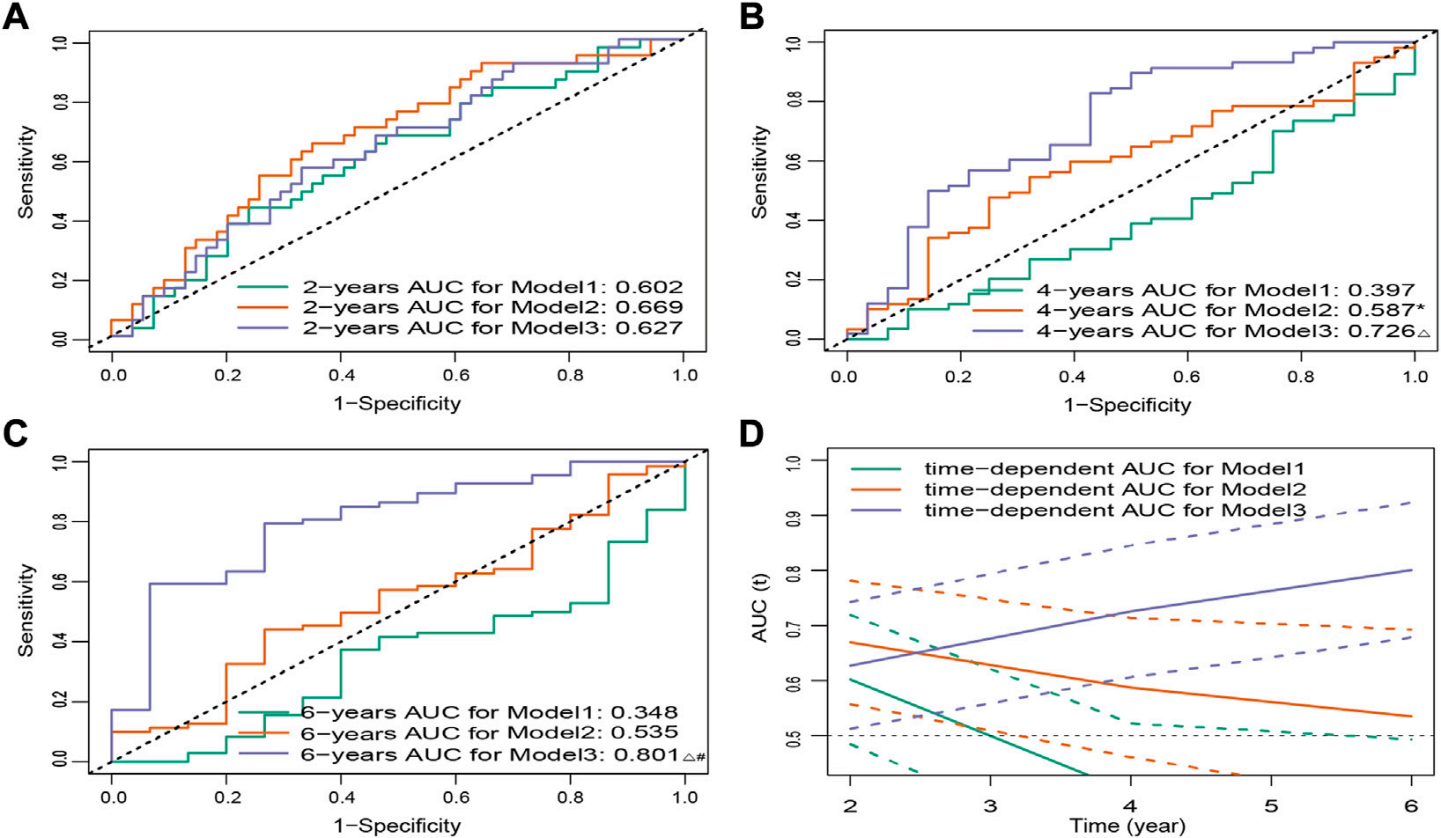
ROC curves for three models of predicting 2- **(A)**, 4- **(B)**, and 6-year **(C)** OS. *p < 0.05 versus Model1, △ *p* < 0.001 versus Model1, #*p* < 0.05 versus Model2. **(D)** Time-dependent ROC curves for three models in the ICGC cohort. Dashed lines represent 95% CIs. ROC, receiver operating characteristic; OS, overall survival; ICGC, International Cancer Genome Consortium; CI, confidence interval.

To illustrate the advantages of established 15-FRG signature, we further compared the performance of our prediction model with that reported previously (Wang H. et al., 2021; Yu et al., 2021) in the ICGC cohort. The results showed that our novel 15-FRG model (Model3) outperformed the 3-gene model (Model1; PMID: 35071242) and 6-gene model (Model2; PMID: 34075060) in predicting overall survival of an individual patient (adj. *p* < 0.05, Figures 5A–C), especially in predicting the long-term survival outcome (OS > 2 years) (Figure 5D).

### Independent prognostic value of the 15-ferroptosis-related gene prognostic signature

**FIGURE 6:**
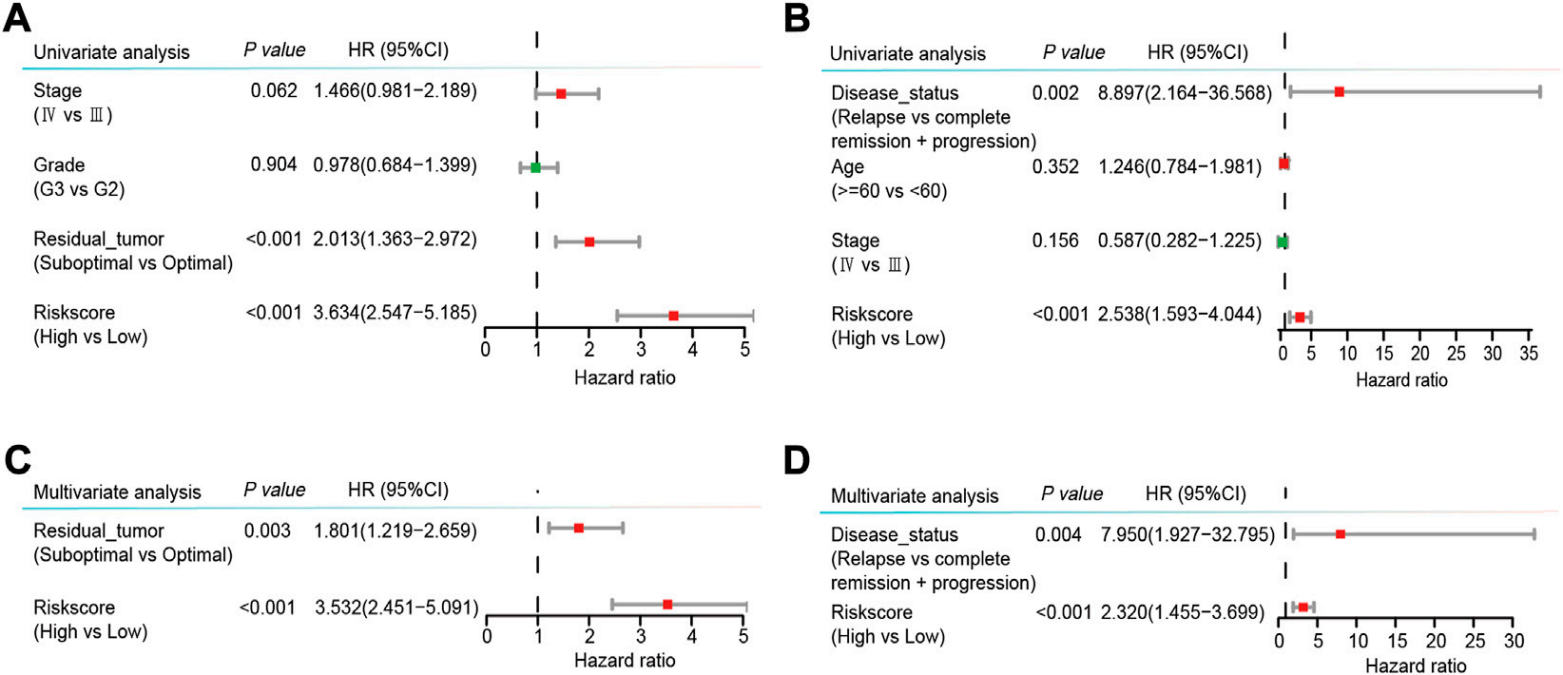
Forest plots of univariable **(A,B)** and multivariable **(C,D)** Cox regression analyses with HRs and 95% CIs in the GSE32062 cohort **(A,C)** and the ICGC cohort **(B,D)**. HR, hazard ratio; CI, confidence interval; ICGC, International Cancer Genome Consortium.

More importantly, we conducted univariate and multivariate Cox regression analyses to explore the independence of the risk score as a prognostic factor of OS. The risk score was found to be obviously related to OS in both the GSE32062 and the ICGC cohorts by univariate Cox regression analysis (HR = 3.634, 95% CI = 2.547–5.185, *p* < 0.001; HR = 2.538, 95% CI = 1.593–4.044, *p* < 0.001, respectively, Figures 6A,B). Furthermore, after the adjustment of the potential confounding factors by multivariate Cox regression analysis, the risk score was validated as an independent prognostic factor of OS in both the GSE32062 and ICGC cohorts (HR = 3.532, 95% CI = 2.451–5.091, *p* < 0.001; HR = 2.320, 95% CI = 1.455–3.699, *p* < 0.001, respectively, Figures 6C,D).

### Prognostic nomogram establishment for predicting the overall survival of ovarian cancer patients

**FIGURE 7:**
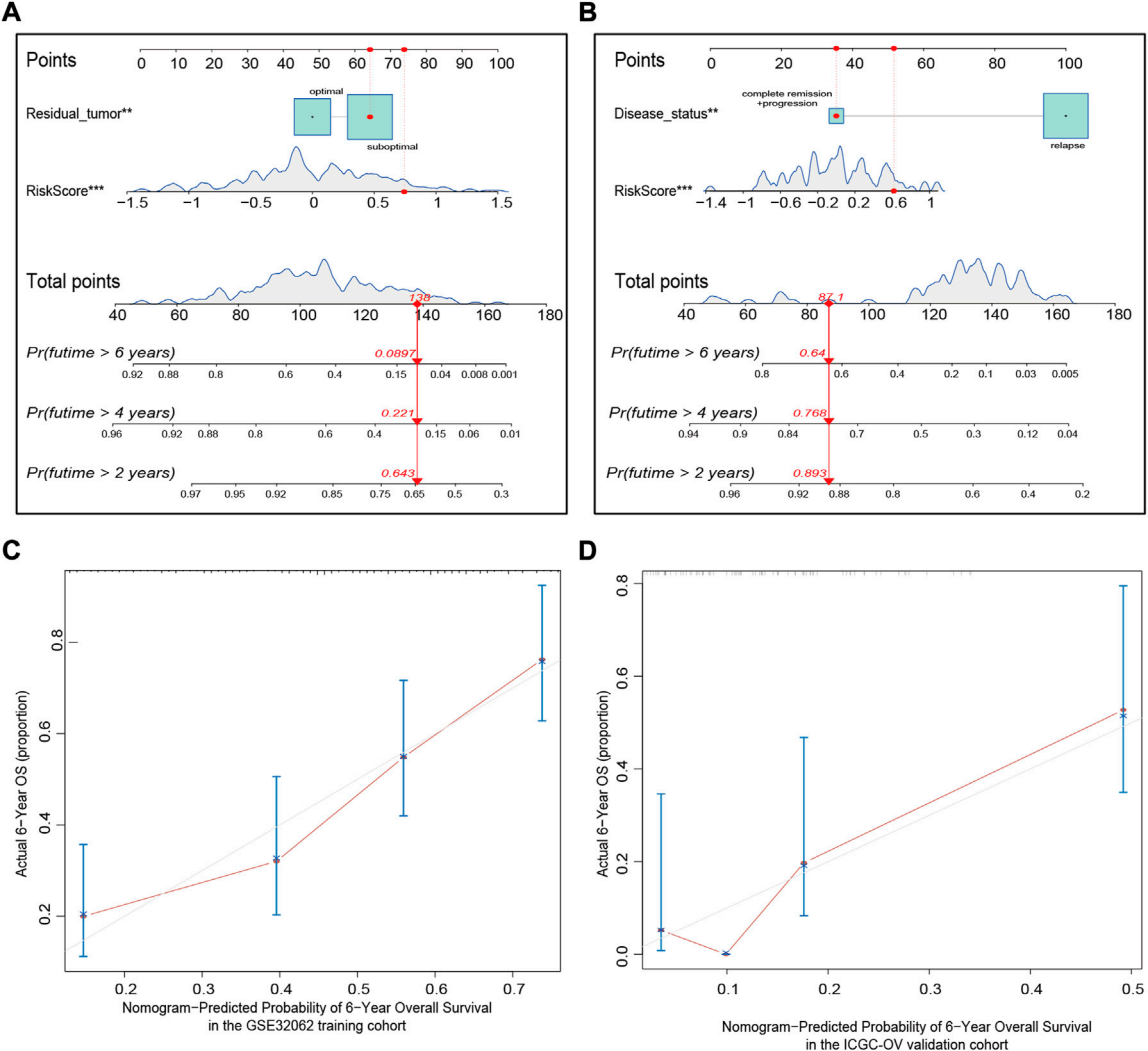
Nomogram establishment for predicting the 2-, 4-, and 6-year OS of OV patients in the GSE32062 **(A)** and ICGC **(B)** cohorts. An example demonstrating the percentage of survival according to the nomogram score is marked in red. To use the nomogram, first find the position of a variable on the variable axis, and then, draw a vertical line upward to find the point number of the variable. The sum of these point numbers is presented on the total points axis, and a vertical line is drawn downward to determine the differential OS probabilities. **p < 0.01; ***p < 0.001. Calibration curves of the nomogram predictive performance in the GSE32062 **(C)** and ICGC **(D)** cohorts. Distributions of the predicted probabilities of 6-year OS are shown at the top of the graphs, the brownish red solid line indicates the performance of the nomogram; the closer the line to the gray line, the better the consistency between predicted and actual outcomes. OS, overall survival; OV, ovarian cancer; ICGC, International Cancer Genome Consortium.

We integrated the available clinical characteristics and risk score into a prediction system to create a novel nomogram system for predicting OS. Statistically significant variables were residual tumor (*p* < 0.01) and risk score (*p* < 0.001) in the GSE32062 cohort model (Figure 7A), and disease status (*p* < 0.01) and risk score (*p* < 0.001) in the ICGC cohort model (Figure 7B). The nomogram demonstrated good predictive accuracy with a C-index of 0.71 (95% CI = 0.66–0.75) in the GSE32062 cohort and 0.65 (95% CI = 0.57–0.72) in the ICGC cohort. Bootstrap validation was performed for calibration of 6-year OS, and the calibration curve indicated excellent agreement between actual and nomogram-predicted outcomes across the spectrum of predictions (Figures 7C,D). Taken together, these results revealed that the nomogram based on 15-FRG signature could efficiently predict patient survival outcome and showed a significant clinical practical value for OV patients.

### Functional analysis of 15-ferroptosis-related gene prognostic signature

**FIGURE 8:**
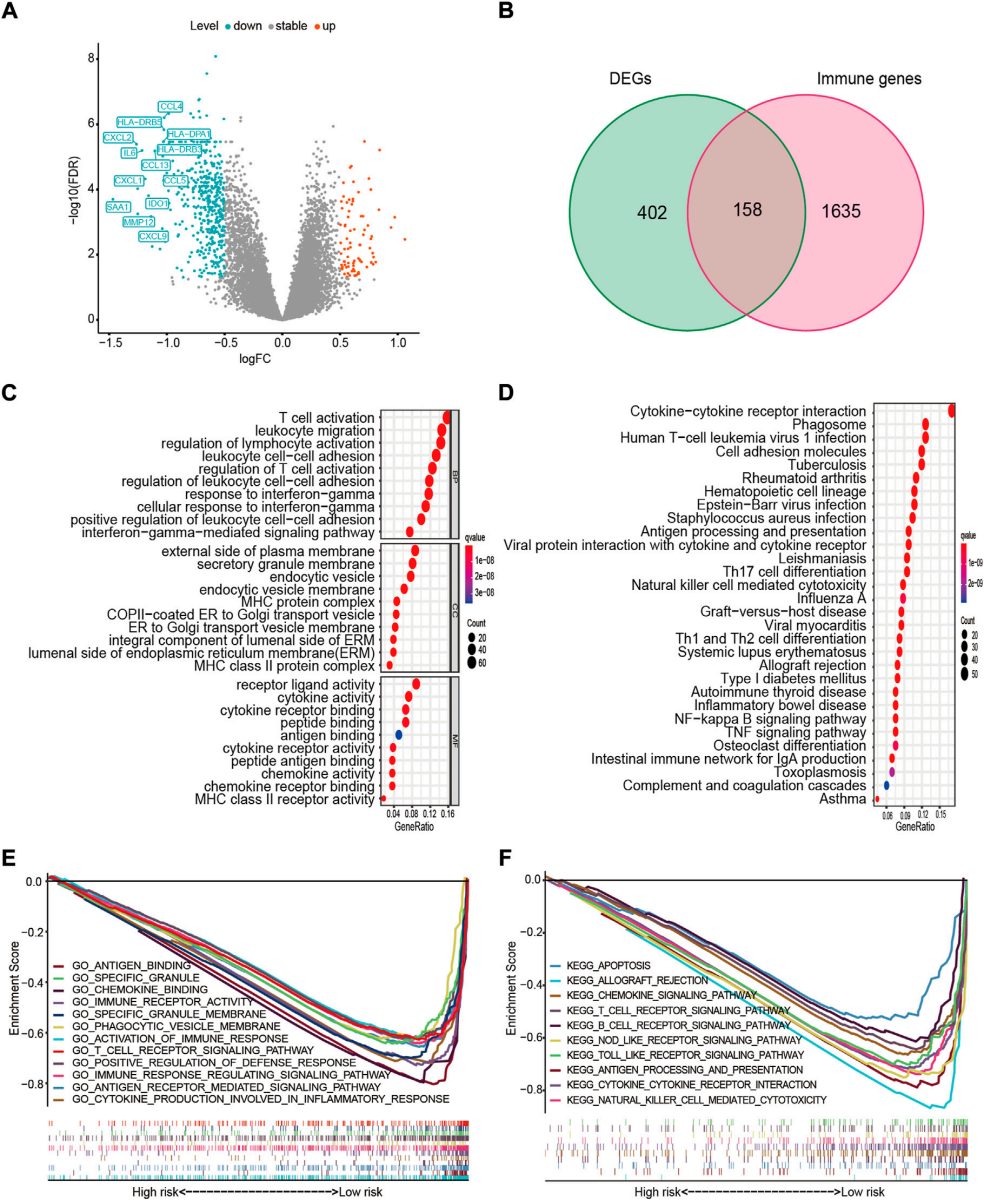
Functional enrichment and pathway analyses. **(A)** Volcano plot of 560 DEGs. Upregulated immune-related DEGs with *p* < 0.05 and |log2FC| > 1 in the low-risk group are presented (left). **(B)** Venn diagram of DEGs and IRGs. **(C)** The top 10 GO terms in molecular function (MF), cellular component (CC), and biological process (BP). **(D)** The top 30 enriched KEGG pathways. **(E)** Most significant GSEA sets, from GO-related MSigDB, associated with the low-risk group. **(F)** Most significant GSEA sets, from KEGG-related MSigDB, associated with the low-risk group. DEG, differentially expressed gene; FC, fold change; IRG, immune-related gene; GO, Gene Ontology; KEGG, Kyoto Encyclopedia of Genes and Genomes; GSEA, gene set enrichment analysis; MSigDB, The Molecular Signatures Database.

To investigate the 15-FRG prognostic signature in terms of underlying biological functions and pathways. GO and KEGG pathway enrichment analyses were performed first for 15 ferroptosis-related genes. The GO and KEGG analysis results indicated that the 15 ferroptosis-related genes were enriched in ferroptosis pathway and lipid-related biologic process, including “regulation of lipid metabolic process” and “regulation of lipid biosynthetic process.” Interestingly, those FRGs were also enriched for immune-related terms, including “positive regulation of MHC class II biosynthetic process,” “positive regulation of T cell differentiation” and “IL−17 signaling pathway” (adj. *p* < 0.05, Supplementary Figures S3A, S3B). In parallel, 560 DEGs were detected between high- and low-risk samples (Figure 8A), and then the GO and KEGG pathway enrichment analyses of 560 DEGs were also performed. As expected, DEGs were also significantly enriched in several iron-related molecular functions, such as “cellular divalent inorganic cation homeostasis,” “response to metal ion,” “divalent inorganic cation transport” and “regulation of reactive oxygen species biosynthetic process” (adj. *p* < 0.05, Supplementary Tables S3A, S3B). Interestingly, among the top 30 GO terms, the DEGs were also obviously enriched in various immune-related GO terms, such as “T-cell activation,” “leukocyte migration,” “regulation of lymphocyte activation,” “leukocyte cell–cell adhesion,” “response to interferon-gamma (IFN-γ),” “major histocompatibility complex (MHC) protein complex,” “cytokine activity” and “antigen binding” (adj. *p* < 0.05, Figure 8C). Meanwhile, some immune-related pathways were also found in the KEGG pathway analysis, including “Cytokine–cytokine receptor interaction,” “Phagosome,” “Antigen processing and presentation,” “Th17 cell differentiation,” “Natural killer cell-mediated cytotoxicity” and “Th1 and Th2 cell differentiation” (adj. *p* < 0.05, Figure 8D).

To further investigate the relationship between the DEGs and immune status, an intersection analysis between the 560 DEGs and 1793 IRGs was performed, revealing 158 immune-related DEGs (Figure 8B). Next, for the high- and low-risk samples, we further conducted GSEA using the molecular signatures database (MSigDB) to elucidate the association between the risk score and immune regulation. GSEA revealed that highly similar immune-related GO terms and KEGG pathways were enriched in the low-risk group (adj. *p* < 0.05, FDR < 0.25, Figures 8E,F), including antigen (“KEGG_ANTIGEN_PROCESSING_AND_PRESENTATION”), cytokine (“KEGG_CYTOKINE_CYTOKINE_RECEPTOR_INTERACTION”), chemokines (“GO_CHEMOKINE_BINDING”), immune cells (“KEGG_T_CELL_RECEPTOR_SIGNALING_PATHWAY”), damage-associated molecular patterns (“KEGG_NOD_LIKE_RECEPTOR_SIGNALING_PATHWAY”; “KEGG_TOLL_LIKE_RECEPTOR_SIGNALING_PATHWAY”), and other immune-regulatory processes. Collectively, these findings suggested that the 15 FRGs and ferroptosis-based risk score were largely associated with immune-related biological processes, and patients in the high- and low-risk group might have different immune landscape.

### Differences in the immune landscape among risk groups

**FIGURE 9:**
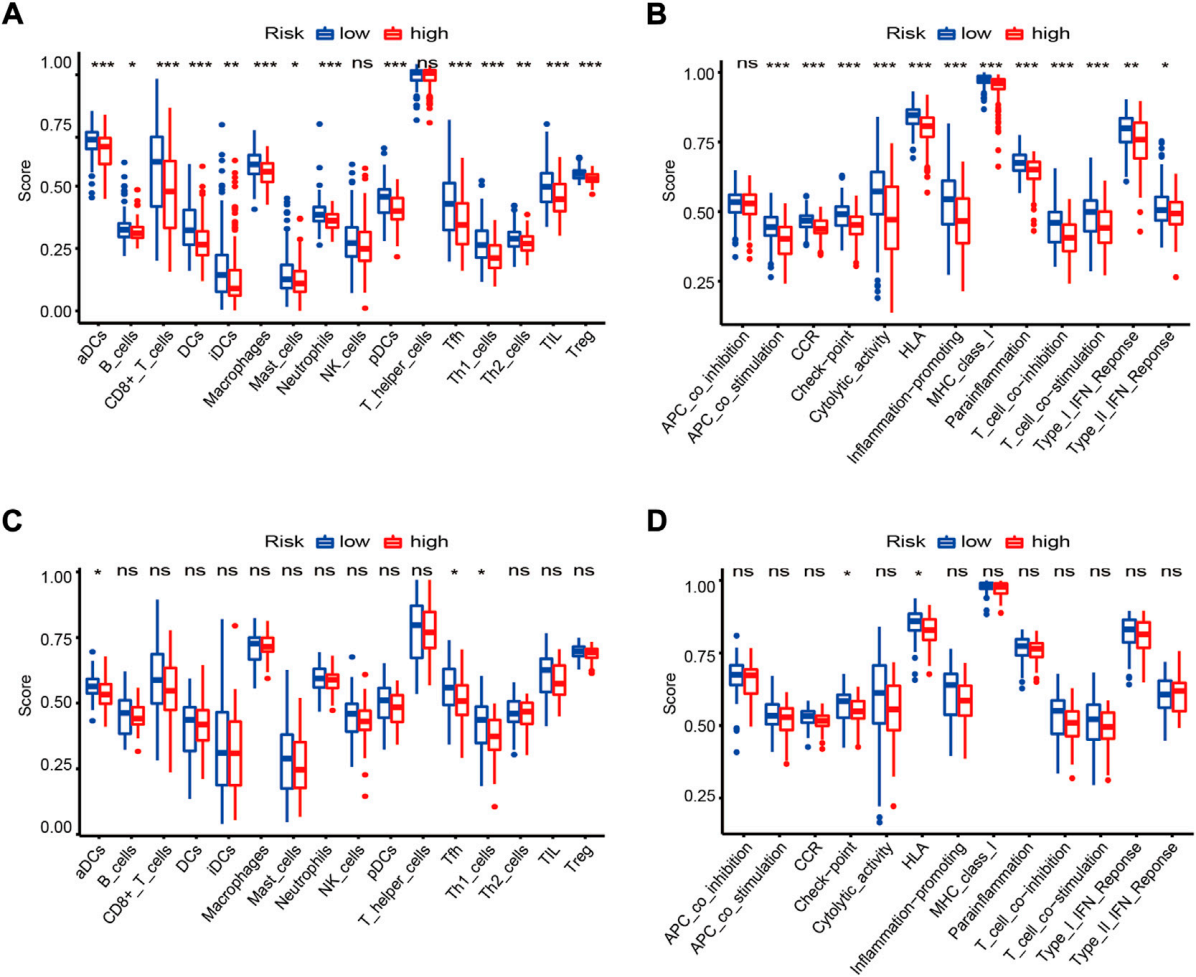
Landscapes of immune cell infiltration and immune-related functions between risk groups in the GSE32062 **(A,B)** and ICGC **(C,D)** cohorts. Boxplots of ssGSEA scores in 16 types of immune cells **(A,C)** and 13 immune-related functions **(B,D)** are presented. *adj. *p* < 0.05; **adj. *p* < 0.01; ***adj. *p* < 0.001; ns, not significant. ICGC, International Cancer Genome Consortium; GSEA, gene set enrichment analysis; CCR, cytokine-cytokine receptor; HLA, human leukocyte antigen.

Given the high correlation between the ferroptosis-based risk score and immune-related biological processes, the relationships among the risk score, immune cell infiltration, and immune-related functions were analyzed in more detail. First, we used the ssGSEA algorithm to assess immune infiltration and immune-related functions in each OV sample in the GSE32062 cohort. The infiltration levels of 14 out of 16 immune cell subpopulations and 12 out of 13 immune-related functions were higher in the low-risk group than in the high-risk group (adj. *p* < 0.05, Figures 9A,B). And then, based on ICGC cohort data, the significant enrichment of three immune cell subpopulations were reconfirmed in the low-risk group, namely antigen-processing and presenting cells subpopulations (“aDCs”) and helper T-cell subpopulations (“Tfh”, “Th1_cells”) (adj. *p* < 0.05, Figure 9C), which were also enriched in the above KEGG analysis. In parallel, the significant enrichment of two immune-related functions were reconfirmed in the low-risk group, namely checkpoint molecules (“Check-point”) and human leukocyte antigen (“HLA”) functions (adj. *p* < 0.05, Figure 9D), corresponding to the GO term “MHC protein complex.”

**FIGURE 10:**
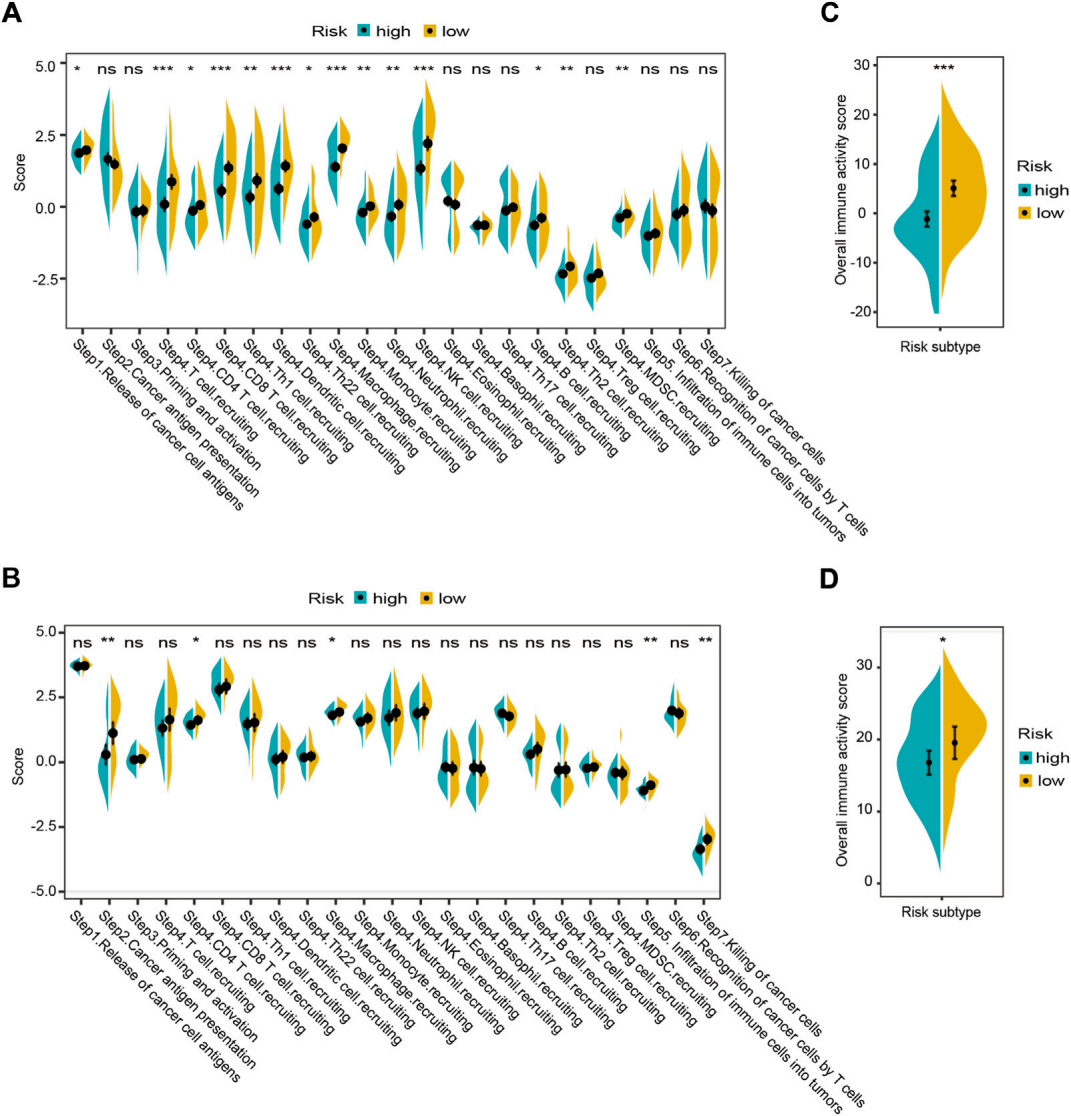
Landscapes of the seven-step cancer-immunity cycle between different risk groups in the GSE32062 **(A,C)** and ICGC **(B,D)** cohorts. Enrichment scores in the seven-step cancer-immunity cycle **(A,B)** and an overall immune activity score **(C,D)** are shown in violin plots. *adj. *p* < 0.05; ** adj. *p* < 0.01; ***adj. *p* < 0.001; ns, not significant. ICGC, International Cancer Genome Consortium.

As the complex antitumor immune response comprises a series of stepwise events (termed cancer-immunity cycle). We determined enrichment scores for the seven-step cancer-immunity cycle using the TIP meta-server tool in both cohorts. The results revealed that antigen release and presentation cycles (“Step 1” and “Step 2”) and immune cells recruitment cycles (“Step 4. CD4 T-cell. recruiting, and Macrophage. Recruiting”) were significantly enriched in the low-risk group (adj. *p* < 0.05, Figures 10A,B). The same trend was also observed for the overall immune activity score of cancer-immunity cycle (by summating the normalized scores of all seven steps) in both cohorts (adj. *p* < 0.05, Figures 10C,D).

**FIGURE 11:**
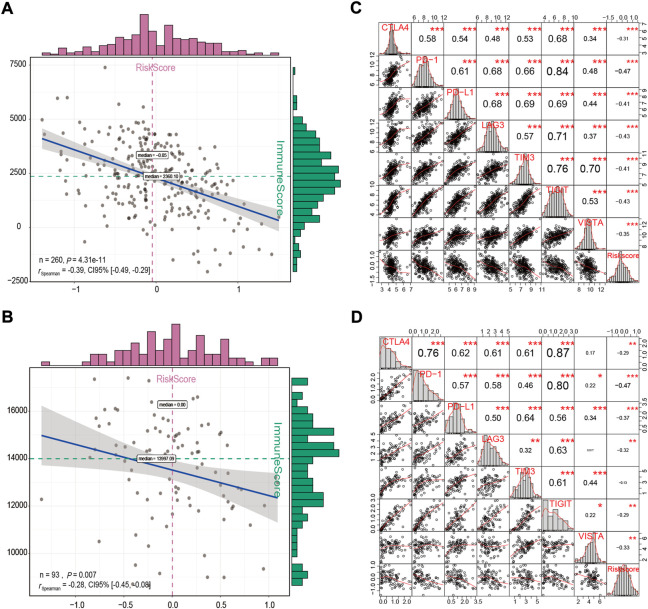
Correlation analysis between the immune score and the risk score **(A,B)**, and between the risk score and the expression levels of immune checkpoint molecules **(C,D)** in the GSE32062 **(A,C)** and ICGC **(B,D)** cohorts. *p < 0.05; **p < 0.01; ***p < 0.001. ICGC, International Cancer Genome Consortium.

A high degree of immune cell infiltration is usually accompanied by high expression levels of immune checkpoint molecules (Galon and Bruni, 2019). It is generally recognized that patients with a high immune score or immune checkpoint expression levels may achieve a higher objective response rate to immunotherapy (Taube et al., 2018). Therefore, the immune score and immune checkpoint molecules have been shown to be predictors of response to various tumor immunotherapies. In this study, we observed a significant negative correlation between the risk score and the immune score in the GSE32062 and ICGC cohorts (Spearman correlation: r = −0.39, *p* < 0.001; r = −0.28, *p* = 0.007, respectively, Figures 11A,B). In addition, we also conducted a correlation analysis between mRNA levels of seven checkpoint molecules and the risk score. The results indicated that most checkpoint molecules were strongly positively correlated with each other, while the mRNA expression levels of CTLA4, PD-1, PD-L1, LAG3, TIGIT, and VISTA were significantly negatively correlated with the risk score in both cohorts (Pearson correlation: r < −0.2, *p* < 0.05, Figures 11C,D). Taken together, the results indicated that patients in the low-risk group had an immune “hot” status, which was characterized by a high degree of immune cell infiltration and multiple checkpoint activation (Galon and Bruni, 2019), and might be more likely to benefit from immunotherapy.

### Prediction performance of the risk score in immunotherapy and chemotherapy

**FIGURE 12:**
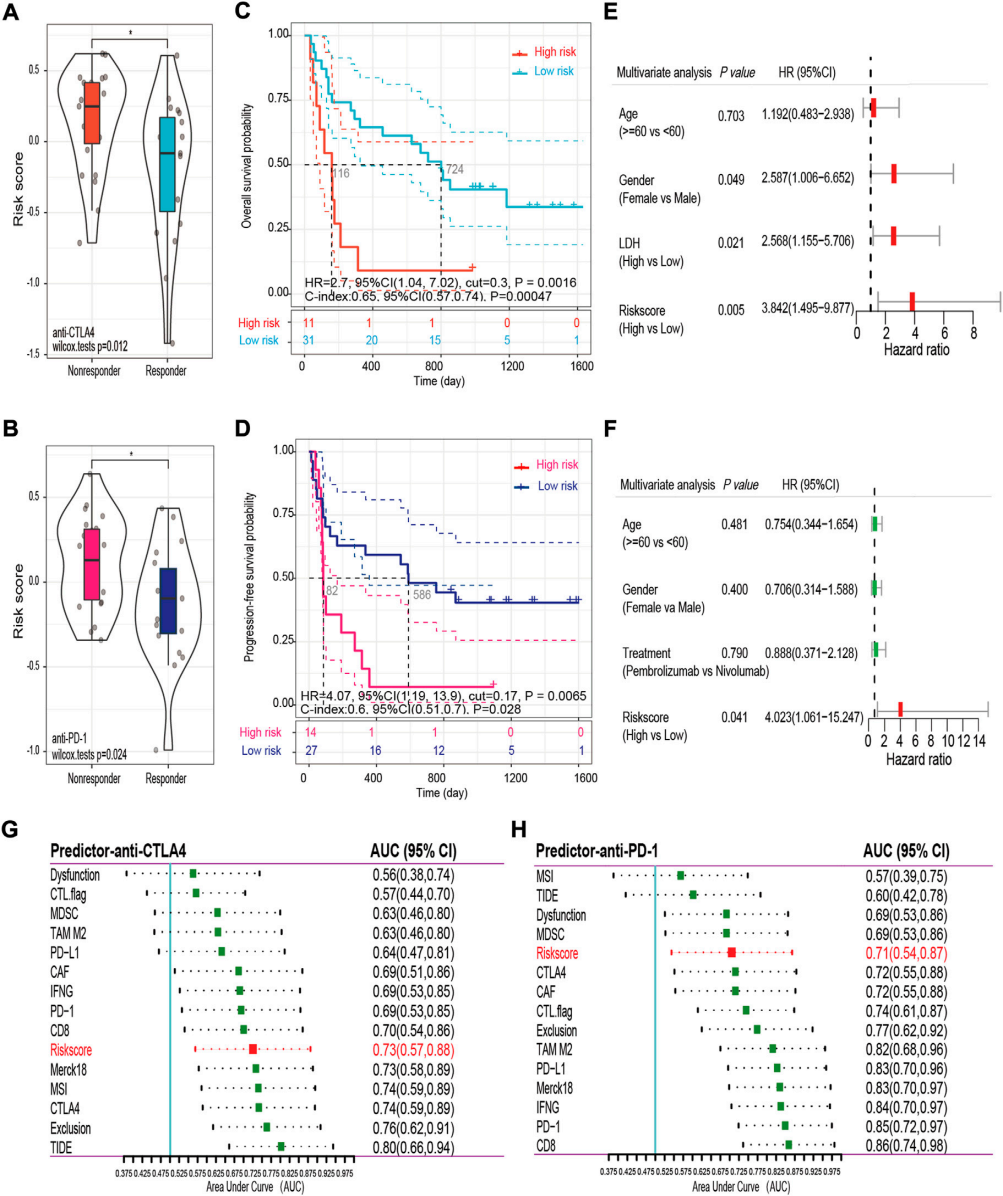
Validation and comparison of the predictive performance of the risk score in the anti-CTLA4 **(A,C,E,G)** and anti-PD-1 **(B,D,F,H)** cohorts. Distributions of the risk scores in the non-responder and responder groups are displayed in violin plots **(A,B)**. Kaplan–Meier curves for OS **(C)** and PFS **(D)** in the different groups. Forest plots of the multivariable Cox regression analysis with HR and 95% CIs **(E,F)**. AUC values and 95% CIs for the 15 predictors are shown in the forest plots **(G,H)**. OS, overall survival; PFS, progression-free survival; HR, hazard ratio; CI, confidence interval; AUC, area under the curve; TIDE: tumor immune dysfunction and exclusion score; IFNG: normalized average expression of IFN-γ response biomarkers, including HLA-DRA, CXCL10, IDO1, STAT1, and IFNG; MSI: microsatellite instability score predicted from gene expression through ridge regression; Merck18: T cell-inflamed signature (PMID: 28650338); PD-L1, PD-1, CTLA4: gene expression values of CD274, PDCD1 and CTLA4; CD8: average expression value of CD8A and CD8B; CTL flag: flag indicator for whether gene expression values are all positive for five cytotoxic T lymphocyte markers, including PRF1, GZMB, GZMA, CD8B, and CD8A; Dysfunction, Exclusion: enrichment scores based on the gene expression signatures of T-cell dysfunction and T-cell exclusion; TAM M2, MDSC, CAF: Pearson correlation coefficients between expression profile and M2 tumor-associated macrophages, myeloid-derived suppressor cells, and cancer-associated fibroblasts.

In spite of the fact that immune checkpoint inhibitors have not yet been approved as routine drugs for patients with OV. We therefore utilized the SubMap algorithm to predict the likelihood of response to immunotherapy in patients with OV. We were very delighted to see that patients with low risk showed a greater likelihood of responding to anti-PD-1 treatment in both OV cohorts (Bonferroni corrected *p* = 0 0.008, Supplementary Figures S3C, S3D). In addition, RNA-seq data and the related clinical information from previous studies, including 42 samples following anti-CTLA4 immunotherapy and 41 samples following anti-PD-1 immunotherapy, were used for external validation. Detailed clinical information for these samples is provided in Supplementary Table S2B. The risk score was calculated for each patients using the 15-FRG risk score formula. First, we observed that the non-responders had a significantly higher risk score than the responders in both external cohorts (*p* = 0.012; *p* = 0.024, respectively, Figures 12A,B). A Kaplan–Meier curve demonstrated that high-risk patients had a significantly lower survival rate than their low-risk counterparts in immunotherapy (Figures 12C,D, *p* < 0.01). Consistently, after adjustment for available confounding factors by multivariate Cox regression, the risk score was still an independent prognostic factor for OS in the anti-CTLA4 cohort and for progression-free survival (PFS) in the anti-PD-1 cohort (HR = 3.842, 95% CI = 1.495–9.877, *p* = 0.005; HR = 4.023, 95% CI = 1.061–15.247, *p* = 0.041, respectively) (Figures 12E,F). Then, we compared the prediction efficiency of the risk score to that of 15 published predictors in both immunotherapy cohorts. Based on sibling comparison between 15 published predictors (Figures 12G,H), we found no significant differences in AUC values among these predictors (*p* > 0.05), the risk score had an AUC value > 0.7 in both immunotherapy cohorts and correlated well with the prediction scores of most predictors (Supplementary Figures S4A, S4B). In contrast, several recently published predictors, such as the TIDE score and MSI, showed significantly performance variations in different immunotherapy regimens (anti-CTLA4 vs. anti-PD-1: 0.80 vs. 0.60; 0.74 vs. 0.57; respectively). In general, these results indicated that the 15-FRG risk score has excellent robustness and generalization ability in predicting the response to different immunotherapy.

In addition to immunotherapy, we also aimed to further understand the chemotherapy comprehensively, the pRRophetic algorithm was used to predict the IC50 of common chemotherapeutic agents in high- and low-risk groups. According to our findings, the estimated IC50 of 45 chemotherapy drugs varied significantly between the high- and low-risk groups, and patients in the low-risk group were more sensitive to the commonly used chemotherapeutic agents, including camptothecin, cytarabine, dasatinib, erlotinib, mitomycin.C and vinblastine (*p* < 0.05, Supplementary Figures S5A–F), which demonstrated that the risk score might serve as a potential predicter of response to chemosensitivity in OV.

## Discussion

In recent years, ferroptosis has attracted much attention, particularly in the area of oncology. Several studies aimed to elucidate the relationship between the ferroptosis-related signature and tumor prognosis, and some progress has been made (Liang et al., 2020; Wang H. et al., 2021). Regretfully, the vast majority of studies have failed to verify these relationships across multiple regions and races, which may lead to weak generalization ability of research results among different races. Therefore, we specifically selected both Asian and Caucasian population datasets as study subjects since the beginning, and then constructed a novel 15-FRG prognostic signature based on the Asian population dataset, which was externally validated for accuracy and robustness based on the Caucasian population. We confirmed that the risk score calculated by the 15-FRG signature was significantly associated with patient overall survival, and could be an independent risk factor for OV prognosis, and showed good prognostic predictive performance in both races. Moreover, the 15-FRG signature was superior to the other two models. Functional analyses indicated that the risk score was closely related to the tumor immunity, and that patients in the high- and low-risk groups exhibited opposite immune landscape. Notably, the risk score might serve as a good predictor of response to immunotherapy and chemotherapy. We supposed that this 15-FRG signature can better assess prognosis and facilitates patient stratification and precision drug treatment in OV.

Previous studies showed that tumor was provoked by multiple genetic mechanisms and key genes. Therefore, using the signature comprised by multiple genes to predict treatment response and prognosis of cancer showed a good clinical application prospect. In our study, we set up a 15-gene prognostic signature with genes screened from ferroptosis in OV. Previous studies regarding other diseases have indicated that these FRGs could be roughly classified into three categories: ferroptosis drivers (CYBB, VDAC2, SOCS1, LINC00472, ELAVL1, IFNG, IDH1), ferroptosis suppressors (NRAS, MT1G, ACSL3, SLC3A2), and ferroptosis markers (PTGS2, SLC1A4, PCK2, XBP1). It has been reported that these 15 genes involve tumorigenesis and tumor development in a variety of cancers. A recent study found that CYBB knockdown decreases ferroptosis and induces chemoresistance *via* the TAZ-ANGPTL4-NOX2 signaling axis in OV (Yang W. et al., 2020). Conversely, CYBB overexpression led to poor prognosis of osteosarcoma (Lin et al., 2021). VDAC2 as a voltage-dependent anion channel was widely explored in multiple FRG prognostic models (Ren et al., 2021; Yi et al., 2021) and bound directly with the ferroptosis activator erastin for increasing the sensitivity of cancer cells to ferroptosis *via* a FOXM1-Nedd4-VDAC2/3 negative feedback loop in melanoma, (Yang Y. et al., 2020). It was well known that the tumor suppressor protein p53 (TP53) was the guardian of the genome that regulated cell survival and death by apoptosis, autophagy, or ferroptosis (Gnanapradeepan et al., 2018). Some FRGs played a p53 context-dependent role in the regulation of ferroptosis. On the one hand, there are FRGs functioning upstream of p53 in ferroptosis, such as SOCS1, which has been found to be sufficient for p53 activation and to reduce SLC7A11 expression and glutathione levels, explaining in part its ability to sensitize cells to a ferroptosis inducer (Saint-Germain et al., 2017). Interestingly, SOCS1 could reduce PD-L1 expression and restore the activation of tumor-infiltrating CD8^+^ T cells, which highlighted its potential as an immune checkpoint inhibitor in OV (Nakagawa et al., 2018). Another p53 upstream molecule was LINC00472, which was significantly correlated with better survival in patients with breast and ovarian cancers (Fu et al., 2016). It mediated apoptosis and ferroptosis in a p53-dependent manner to suppress cancer progression by interacting with Ras GTPase-activating protein-binding protein 1 (Mao et al., 2018). On the other hand, there are FRGs functioning downstream of p53 in ferroptosis. For example, ELAVL1, which was negatively regulated by miR-139-3p (Xue et al., 2019), was associated with poor prognosis and contributes to invasion, migration, and cell proliferation in OV (Huang et al., 2016). PTGS2, which encoded cyclooxygenase-2 (COX-2), was upregulated by the ferroptosis agonist erastin only in p53 wild-type cells, suggesting that its regulation was p53-dependent. Meanwhile, prognostic analysis suggested that higher PTGS2 expression may be associated with poor OS in OV, but the results of different studies were somewhat conflicting (Steffensen et al., 2007). Moreover, SLC1A4, PCK2 and XBP1 were upregulated by 2-fold in erastin-treated HT-1080 cells (Dixon et al., 2014) and could be used as powerful prognostic markers in hepatobiliary cancer (Liu et al., 2020; Peng et al., 2021; Wang et al., 2022). As the key genes of ferroptosis, SLC3A2, MT1G and ACSL3 have also been widely explored in multiple FRG prognostic models. SLC3A2 was suppressed by IFN-γ that produced by activated CD8^+^ T cells, which resulted in a restriction of cystine uptake and then enhanced tumor lipid peroxidation and ferroptosis, and improved tumor control (Lang et al., 2019; Wang W. et al., 2019). MT1G as a critical regulator of sorafenib resistance could inhibit sorafenib-induced ferroptosis in hepatocellular carcinoma through decreased glutathione depletion and lipid peroxidation (Sun et al., 2016). ACSL3, which was required for exogenous monounsaturated fatty acid activation, promoted a ferroptosis-resistant cell state (Magtanong et al., 2019). It is well known that gene mutation may cause splicing changes, resulting in altered gene function or altered pathways. Therefore, mutant FRGs may play a dual role in ferroptosis. Oncogenic mutant NRAS protected cells from oxidative stress-induced ferroptosis in primary rhabdomyosarcoma (Schott et al., 2015), whereas wild-type NRAS appeared to do the opposite. Wild-type IDH1 was an NADP + -dependent protein that catalyzed the production of NADPH from NADP+, which in turn sustained lipid biosynthesis and redox homeostasis in the TCA cycle (Sonego and Baldassarre, 2020). Conversely, mutant IDH1 could break the homeostasis and promote ROS accumulation, and sensitize cells to ferroptosis through a reduction in glutathione peroxidase 4 (GPX4), a core enzyme in lipid ROS scavenging and ferroptosis (Wang T. et al., 2019). In addition, through the literature review, we found that except CYBB, VDAC2, IDH1, MT1G, SLC1A4, PCK2, SLC3A2, the prognostic value of other FRGs in ovarian cancer has been reported, which provided a possibility for constructing a prognostic model. Meanwhile, our study reconfirmed that these genes were closely related to OS in OV, and used these 15 FRGs to construct a novel prognostic signature. The predictive power of the risk score calculated by the signature was proved to be reliable in different ethnic groups, and the performance was even superior to some of the reported prognostic risk models. Moreover, the risk score was an independent risk factor for OV patients, and patients in the low-risk group showed longer OS and better prognosis. We believed that these results could help to implement stratified management of ovarian cancer patients.

Notably, functional analysis revealed a broad immune-related functional spectrum based on DEGs between the high- and low- risk groups. It was reasonable to assume that there was a close correlation between the risk score and tumor immunity. Therefore, we used various immune profile-relevant analytical methods to gain additional insights into the immune landscape. The results indicated that patients with a high-risk score were in an immune “cold” phenotypic state, with low levels of immune cell infiltration (e.g., CD4^+^) and cancer-immunity cycle steps as well as reduced antigen-presenting capacity (e.g., aDCs). One possible hypothesis was that activated immune infiltrating cells enhanced ferroptosis-specific lipid peroxidation in OV cells, and that, in turn, the increased numbers of ferroptotic cells released distinct tumor-associated antigens to further attract immune cell infiltration. This interaction network between ferroptosis and immune infiltrating cells, analogous to damage-associated molecular patterns (DAMPs) (Garg and Agostinis, 2017), may ultimately contribute to the antitumor efficacy of ferroptosis (Wang W. et al., 2019). Therefore, the immune “cold” state in patients with a high-risk score may explain their poor prognosis. Based on this assumption, it can be envisaged that strategies that combine immunotherapies with classical chemotherapies promoting ferroptosis may turn immune “cold” (high-risk) tumors into “hot” (low-risk) tumors, which will help to improve patient prognosis.

Up to this point, treatment options remain limited in OV with high rates of recurrence and chemoresistance. Immunotherapy, as one of the frontiers of tumor therapy, has been receiving increasing attention from gynecologists. Despite this, clinical research on anti-PD-1 and PD-L1 immune checkpoint blockade has shown that only a limited percentage of patients exhibit a durable clinical benefit (Matulonis et al., 2019). Therefore, early identification of patients with potential response to the immunotherapy was crucial to improving their prognosis. Regrettably, predictors of the response to immunotherapy were scarce. It was reported that immune checkpoint molecules and immune score might be potential predictive biomarkers for the efficacy of immunotherapy (Fu et al., 2020). Interestingly, we found that the ferroptosis-based risk score was negatively related with various immune checkpoint biomarkers and the immune score, highlighting that risk score could be a predictor and low-risk patients might more specifically benefit from immune checkpoint blockade-based immunotherapies. The submap results also suggested that patients with low risk might have a high likelihood of responding to anti-PD1 immunotherapy. Meanwhile, multiple immune checkpoint biomarkers were positively correlated with each other, suggesting that multi-target immunotherapy may overcome the resistance to single-target immunotherapy. Moreover, by comparing with 14 well-validated predictors (Fu et al., 2020), including the classical biomarkers PD1, PDL-1, TIDE, and MSI, we validated that the prediction accuracy and robustness of the risk score performed well. However, these findings were made in melanoma datasets and further clinical verification is required in patients with OV. Ovarian cancer is usually treated with platinum-based chemotherapy. Using the pRRophetic algorithm, we imputed that patients with low risk could be more sensitive to some commonly used chemotherapeutic agents. These results indicated that the risk score might improve our understanding of immunotherapy and facilitate a precise application of immunotherapy and chemotherapy in cancer patients.

There are several key limitations of our analyses. First, the signature was built and validated using retrospective samples, validation using prospective real-world samples was also required. In addition, the hallmark genes evaluated in our study were restricted to FRGs. Hence, the intrinsic weakness of predictive power was inevitable. Further, the associations between the risk score and the immune landscape were estimated by bioinformatics analysis, and we did not conduct further experimental verification.

## Conclusion

In conclusion, our study constructed a novel 15-FRG prognostic signature that performed well in Asian and Caucasian populations, and might serve as an effective predictor of response to immunotherapy. Our findings may provide a better insight into OV prognostic management and may serve as a basis to facilitate a precise application of immunotherapy in OV. The underlying mechanisms between FRGs and the immune microenvironment in OV remained to be investigated.

## Data Availability

Publicly available datasets were analyzed in this study. This data can be found here: http://tide.dfci.harvard.edu/, https://dcc.icgc.org/, https://www.ncbi.nlm.nih.gov/geo/query/acc.cgi?acc=GSE32062.

## Supplementary material

SUPPLEMENTARY FIGURE S1 Stratified Kaplan–Meier survival curves. Kaplan–Meier survival curves stratified by tumor stage **(A)**, tumor grade **(B)**, and residual tumor **(C)** in the GSE32062 cohort. Kaplan–Meier survival curves stratified by age **(D)**, tumor stage **(E)**, and disease status **(F)** in the ICGC cohort.

SUPPLEMENTARY FIGURE S2 Kaplan–Meier survival curves of 15 FRGs constructed using a Kaplan–Meier plotter tool (http://www.kmplot.com). Auto select best cutoff was chosen in the analysis. FRG, ferroptosis-related gene.

SUPPLEMENTARY FIGURE S3 The top 10 enriched GO terms related to 15-FRGs in molecular function (MF), cellular component (CC), and biological process (BP) **(A)**. The top 14 enriched KEGG pathways related to 15-FRGs **(B)**. Immunotherapeutic responses to anti-CTLA-4 and anti-PD-1 treatments in the GSE32062 **(C)** and ICGC **(D)** cohorts. GO, Gene Ontology; KEGG, Kyoto Encyclopedia of Genes and Genomes. FRG, ferroptosis-related gene.

SUPPLEMENTARY FIGURE S4 Correlations between the risk score and other predictor scores in the anti-CTLA4 **(A)** and anti-PD-1 **(B)** cohorts. **p* < 0.05; ***p* < 0.01; ****p* < 0.001. TIDE: tumor immune dysfunction and exclusion score; IFNG: normalized average expression of IFN-γ response biomarkers, including HLA-DRA, CXCL10, IDO1, STAT1, and IFNG; MSI: microsatellite instability score predicted from gene expression through ridge regression; Merck18: T cell-inflamed signature (PMID: 28650338); PD-L1, PD-1, CTLA4: gene expression values of CD274, PDCD1 and CTLA4; CD8: average expression value of CD8A and CD8B; CTL flag: flag indicator for whether gene expression values are all positive for five cytotoxic T lymphocyte markers, including PRF1, GZMB, GZMA, CD8B, and CD8A; Dysfunction, Exclusion: enrichment scores based on the gene expression signatures of T-cell dysfunction and T-cell exclusion; TAM M2, MDSC, CAF: Pearson correlation coefficients between expression profile and M2 tumor-associated macrophages, myeloid-derived suppressor cells, and cancer-associated fibroblasts.

SUPPLEMENTARY FIGURE S5 Estimated IC_50_ values of six commonly used chemotherapeutic agents for high- and low-risk groups in the GSE32062 cohort **(A–F)**.

SUPPLEMENTARY TABLE S1 List of the ferroptosis-related genes (A) immune-related genes (B) immune infiltration annotated gene sets (C).

SUPPLEMENTARY TABLE S2 Characteristics of patients in the GSE32062 and ICGC-OV-AU cohorts (A) and the anti-CTLA4 and anti-PD-1 cohorts (B).

SUPPLEMENTARY TABLE S3 Table of GO terms (A) and KEGG pathways (B) significantly enriched in 15 ferroptosis-related genes.
