# Supplementary figures and images for: Identification of a novel ferroptosis-related gene signature associated with prognosis, the immune landscape, and biomarkers for immunotherapy in ovarian cancer
Source: Front Pharmacol. 2022 Oct 25;13:949126. doi: 10.3389/fphar.2022.949126 (PMC9641277; doi:10.3389/fphar.2022.949126)

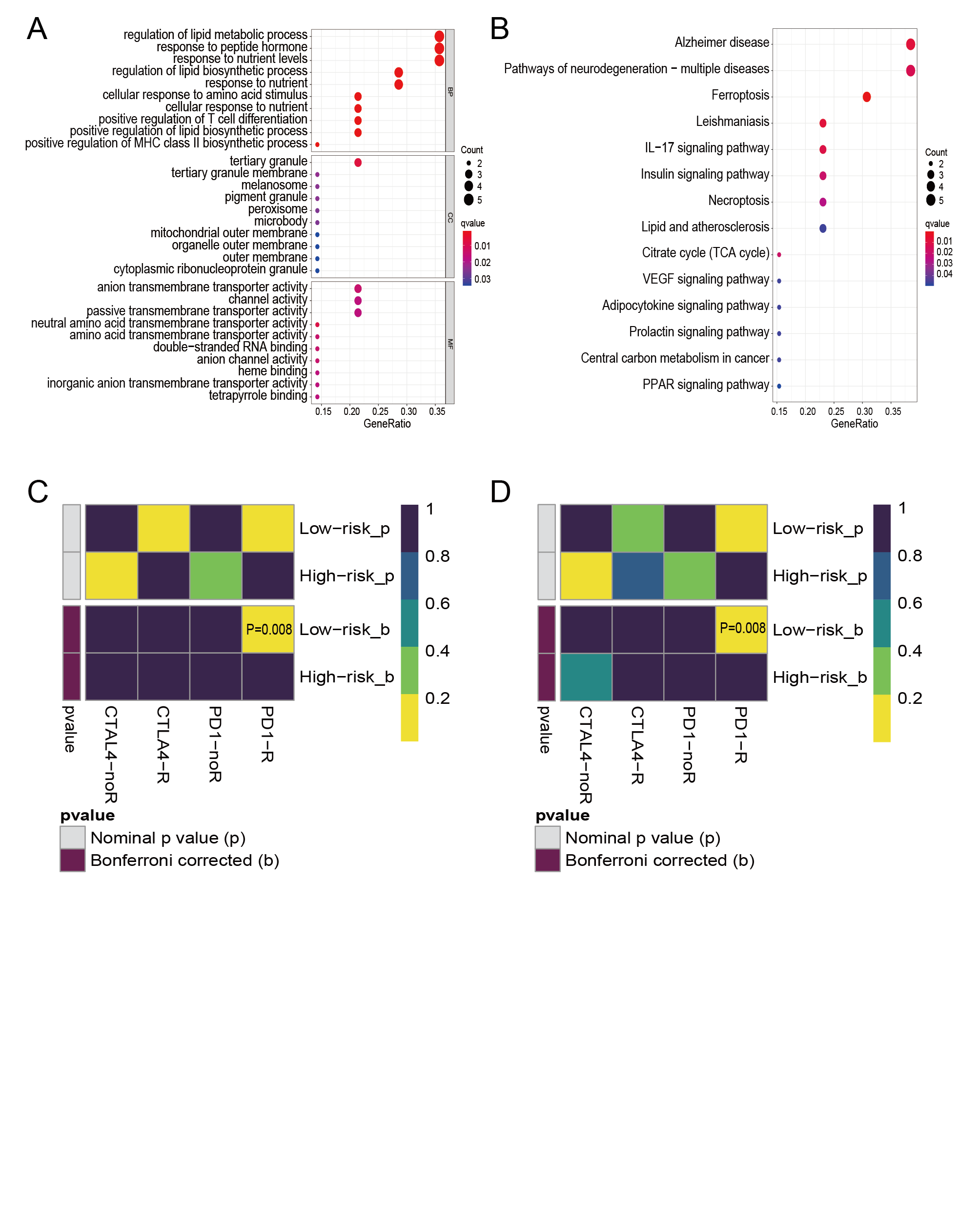

Supplement: Supplementary file 1 [file Image3.tif]

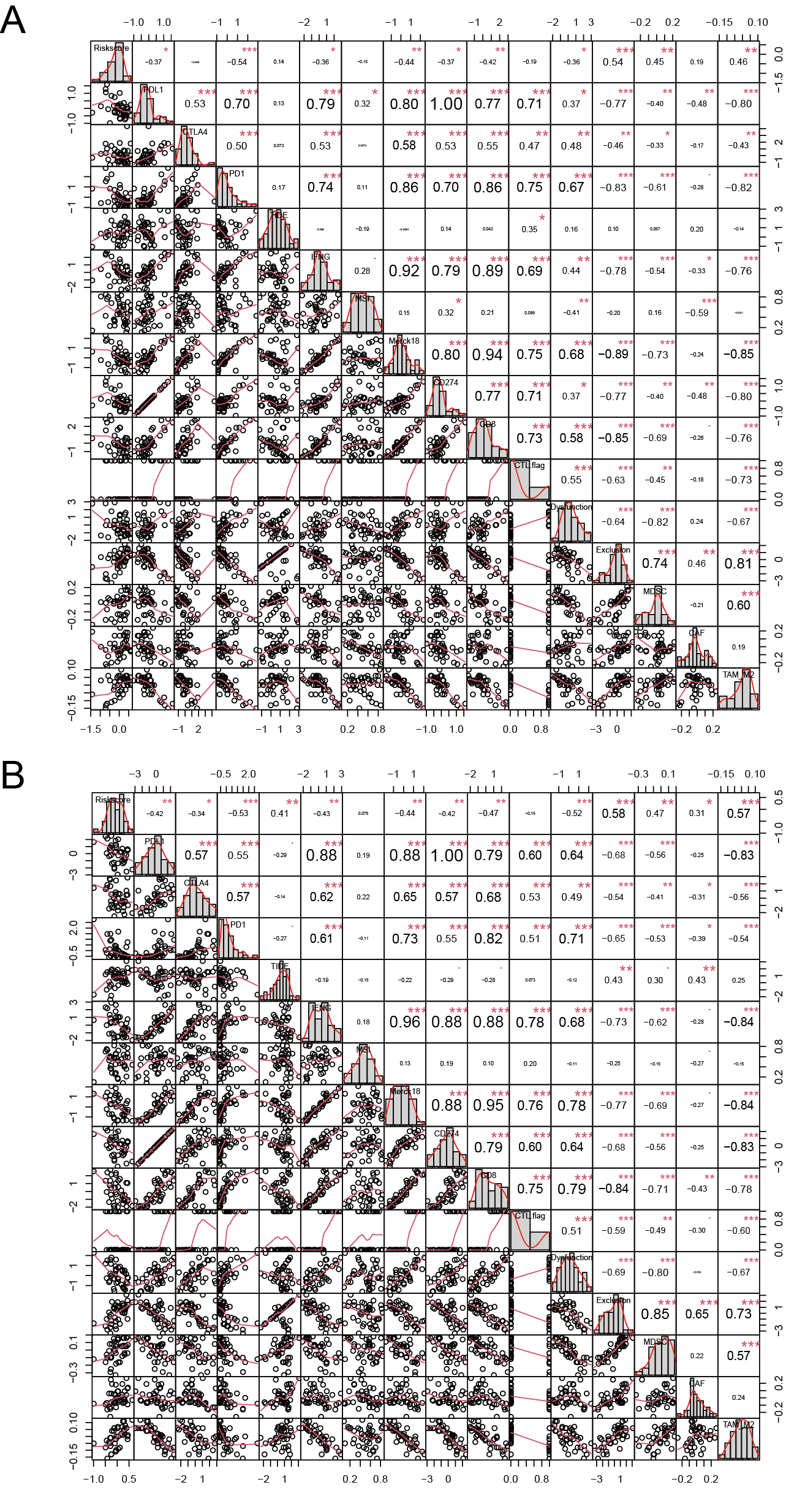

Supplement: Supplementary file 2 [file Image4.tif]

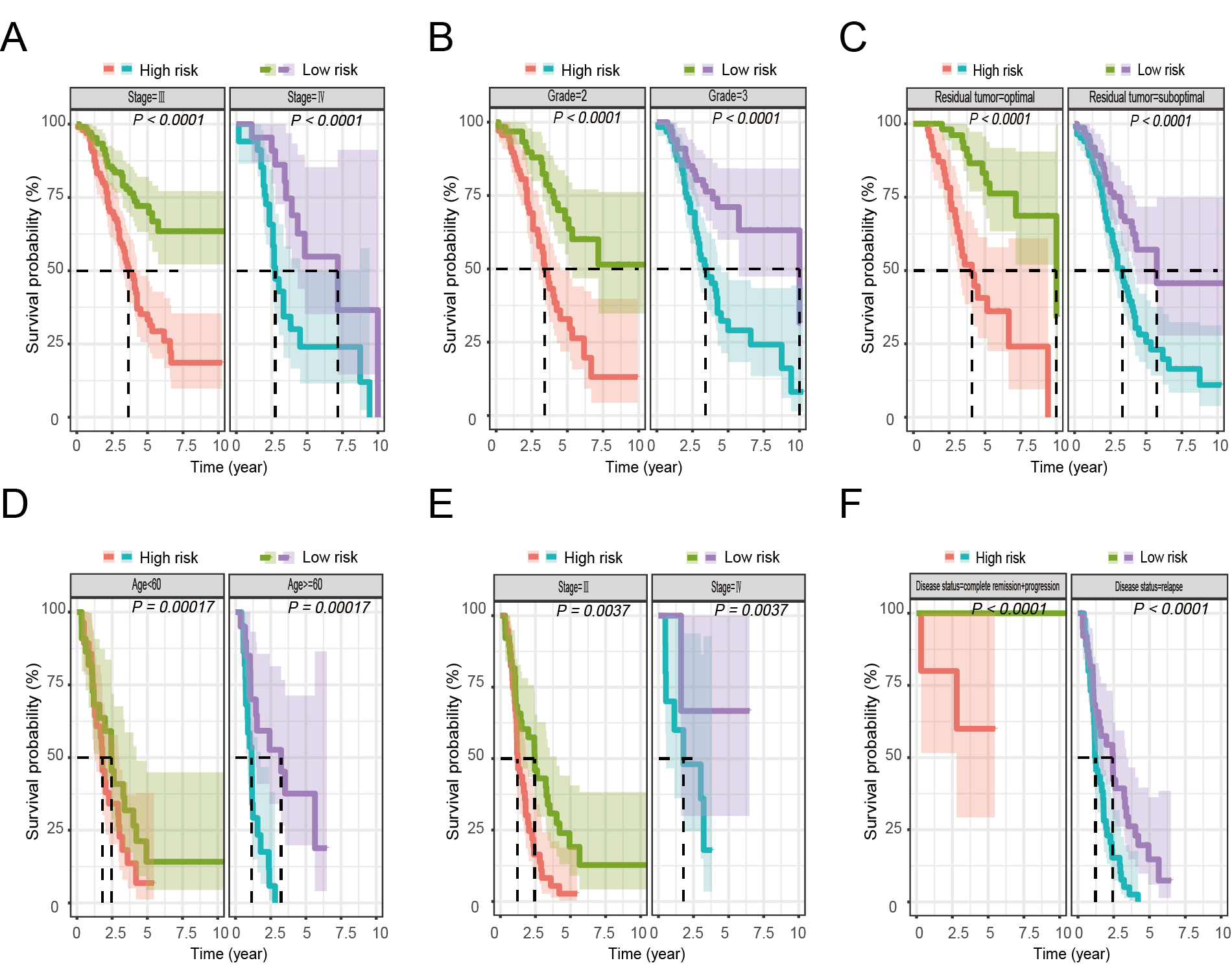

Supplement: Supplementary file 3 [file Image1.tif]

A

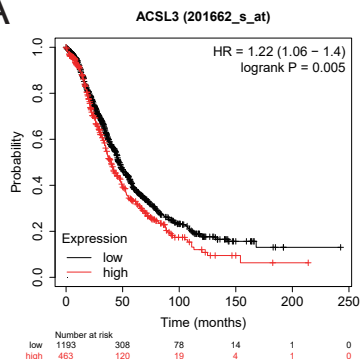

B

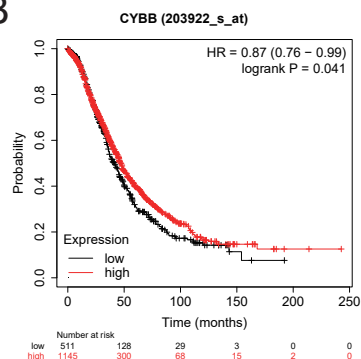

C

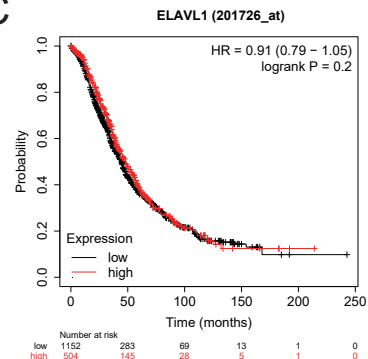

D

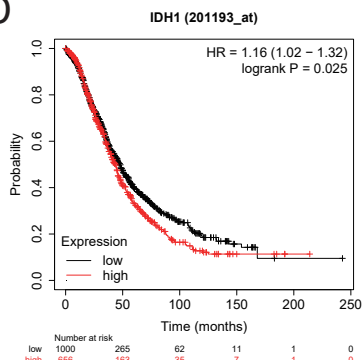

E

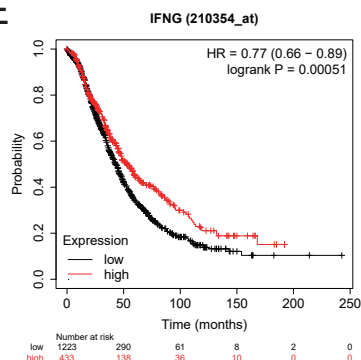

F

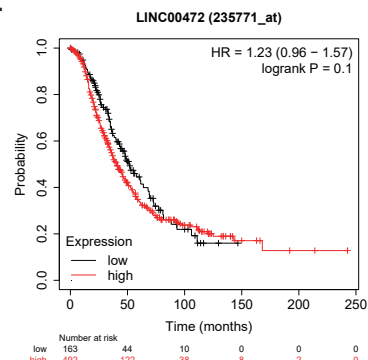

G

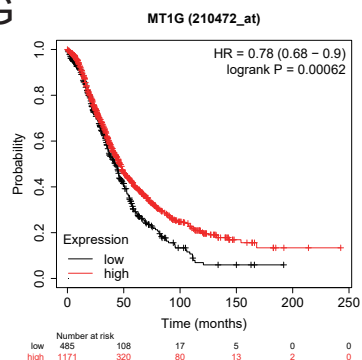

H

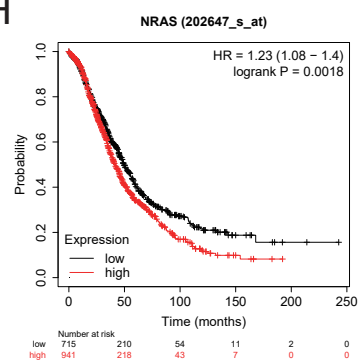

I

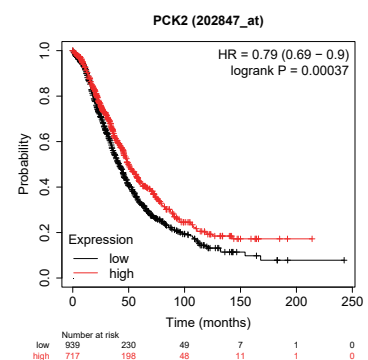

J

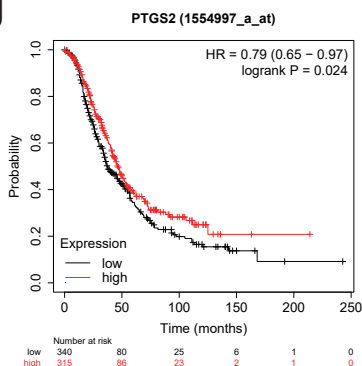

K

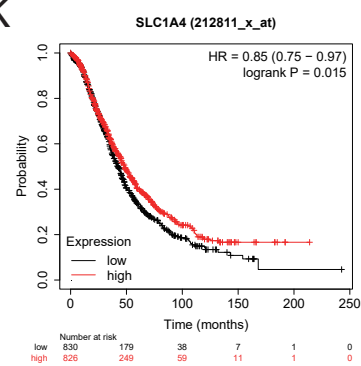

L

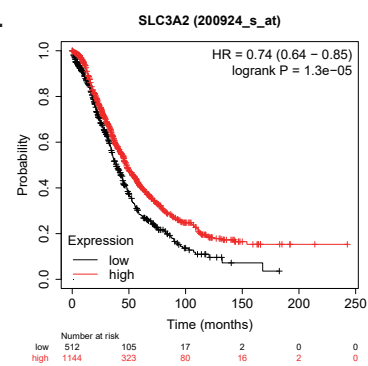

M

SOCS1 (209999\_x\_at)

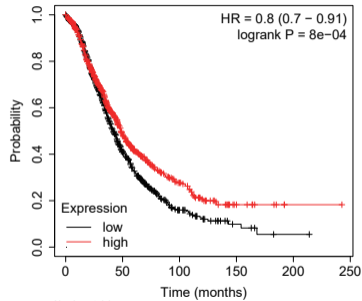

N

VDAC2 (211662\_s\_at)

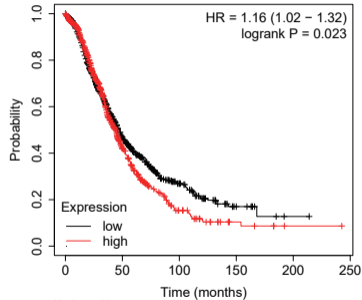

O

XBP1 (200670\_at)

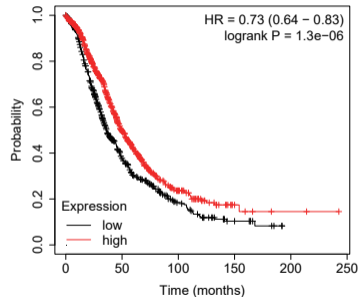

Supplement: Supplementary file 4 [file Image2.pdf]

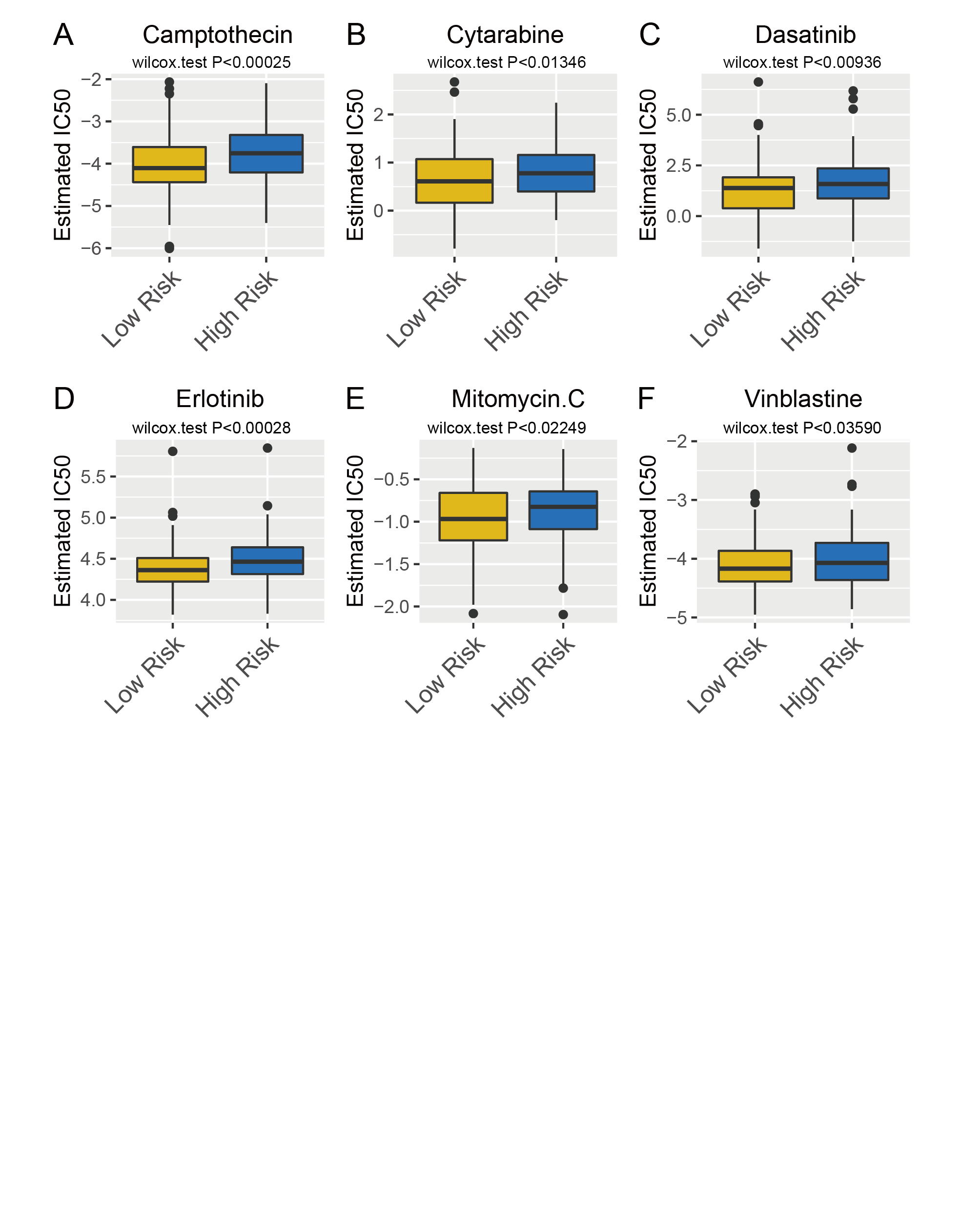

Supplement: Supplementary file 6 [file Image5.tif]
